# LC3-associated phagocytosis in bone marrow macrophages suppresses acute myeloid leukemia progression through STING activation

**DOI:** 10.1172/JCI153157

**Published:** 2022-03-01

**Authors:** Jamie A. Moore, Jayna J. Mistry, Charlotte Hellmich, Rebecca H. Horton, Edyta E. Wojtowicz, Aisha Jibril, Matthew Jefferson, Thomas Wileman, Naiara Beraza, Kristian M. Bowles, Stuart A. Rushworth

**Affiliations:** 1 Norwich Medical School, University of East Anglia, Norwich, United Kingdom.; 2 Earlham Institute, Norwich, United Kingdom.; 3 Department of Haematology, Norfolk and Norwich University Hospitals NHS Trust, Norwich, United Kingdom.; 4 Quadram Institute, Norwich, United Kingdom.

**Keywords:** Hematology, Oncology, Autophagy, Leukemias, Mitochondria

## Abstract

The bone marrow (BM) microenvironment regulates acute myeloid leukemia (AML) initiation, proliferation, and chemotherapy resistance. Following cancer cell death, a growing body of evidence suggests an important role for remaining apoptotic debris in regulating the immunologic response to and growth of solid tumors. Here, we investigated the role of macrophage LC3–associated phagocytosis (LAP) within the BM microenvironment of AML. Depletion of BM macrophages (BMMs) increased AML growth in vivo. We show that LAP is the predominate method of BMM phagocytosis of dead and dying cells in the AML microenvironment. Targeted inhibition of LAP led to the accumulation of apoptotic cells (ACs) and apoptotic bodies (ABs), resulting in accelerated leukemia growth. Mechanistically, LAP of AML-derived ABs by BMMs resulted in stimulator of IFN genes (STING) pathway activation. We found that AML-derived mitochondrial damage–associated molecular patterns were processed by BMMs via LAP. Moreover, depletion of mitochondrial DNA (mtDNA) in AML-derived ABs showed that it was this mtDNA that was responsible for the induction of STING signaling in BMMs. Phenotypically, we found that STING activation suppressed AML growth through a mechanism related to increased phagocytosis. In summary, we report that macrophage LAP of apoptotic debris in the AML BM microenvironment suppressed tumor growth.

## Graphical abstract

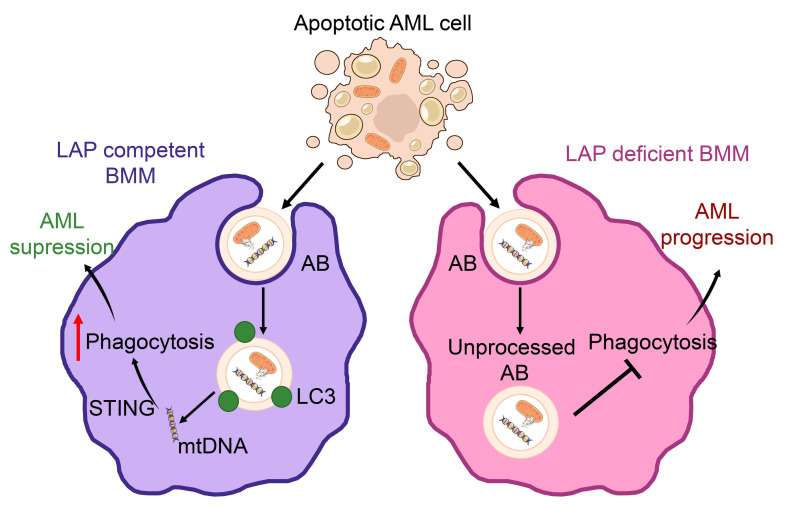

## Introduction

Acute myeloid leukemia (AML) is a genetically, biologically, and clinically heterogeneous set of diseases that share in common the malignant proliferation of clonal hematopoietic stem and progenitor cells (HSPCs) within the bone marrow (BM) microenvironment (1). In a minority of patients with AML, a cure can be achieved that is medically effected either through intensive cytotoxic chemotherapy or, alternatively, via an alloimmune-mediated graft-versus-leukemia mechanism. Curiously, for such a clinically aggressive, and often chemo-refractory, disease, ex vivo AML exhibits a high level of spontaneous apoptosis (2–6). Furthermore, relapse for patients who achieve therapy-induced remission occurs from minimal residual disease sequestered within the BM microenvironment (7–9). Taken together, these observations illustrate the fundamental importance of the BM microenvironment in leukemia initiation, proliferation, and chemoresistance across the broad set of AML subtypes.

The BM microenvironment is a complex, highly organized organ evolved to support the life-long production of blood cells from hematopoietic stem cells (HSCs). HSCs reside within niches, where their fate is mediated through interactions with multiple hematopoietic and nonhematopoietic cells, regulated by direct cell-cell contact, growth factors, and cytokines (10–14). BM macrophages (BMMs) have long been recognized to have diverse and indispensable, tissue-specific roles in host defense and tissue homeostasis (15, 16). In the BM, macrophages contribute to the maintenance of the HSC niche, and their depletion results in the egress of HSCs into the peripheral blood (17). In addition, BMMs are involved in processes regulating HSC quiescence and have been reported to negatively regulate the HSC pool in response to infection (18, 19). In cancer, protumoral tumor-associated macrophages (TAMs) appear to be fundamentally involved in cancer progression and metastasis and are linked to poorer clinical outcomes across a diverse spectrum of malignancies and tumor microenvironments (20–26). In AML, macrophages have been shown to interact with leukemic cells to promote AML progression through mechanisms of action linked to phagocytosis and immune modulation (27–29). Expression of the transmembrane protein CD47 correlates with a poor prognosis in patients with AML, in part through inhibition of mononuclear phagocytosis of leukemia stem cells (LSCs). In vivo experiments using monoclonal antibodies to block AML cell-surface CD47 interaction with SIRPα on macrophages enables the phagocytosis of LSCs (30). This work, which has since been extended into clinical trials (NCT04778397 and NCT04912063; refs. 31, 32), provides a proof-of-concept paradigm for the therapeutic modulation of macrophage phagocytic function in the AML microenvironment.

Phagocytosis by mononuclear cells occurs either by an LC3-dependent or -independent, mechanism. LC3-associated phagocytosis (LAP) is physiologically triggered by pathogens and cellular debris via interaction with phagocyte surface receptors, including T cell membrane protein 4 (TIM-4) and FcR. This results in a rapid response to phagosomal maturation and degradation of cargo (33). Although LAP shares components similar to those in canonical autophagy, LAP has been shown to be a distinct process (34). Impairment of LAP in myeloid cells has been shown to suppress solid tumor growth (35), however, the tumor-specific roles and functions of LAP in blood cancers have not, to our knowledge, been defined. In the present study, we investigate the role of LC3-mediated phagocytosis in the context of AML. We examine the mechanisms regulating BMM LAP in the leukemia microenvironment and the outcomes of targeting the LAP pathway in models of AML.

## Results

### Depletion of phagocytes increases AML tumor burden.

**Figure 1:**
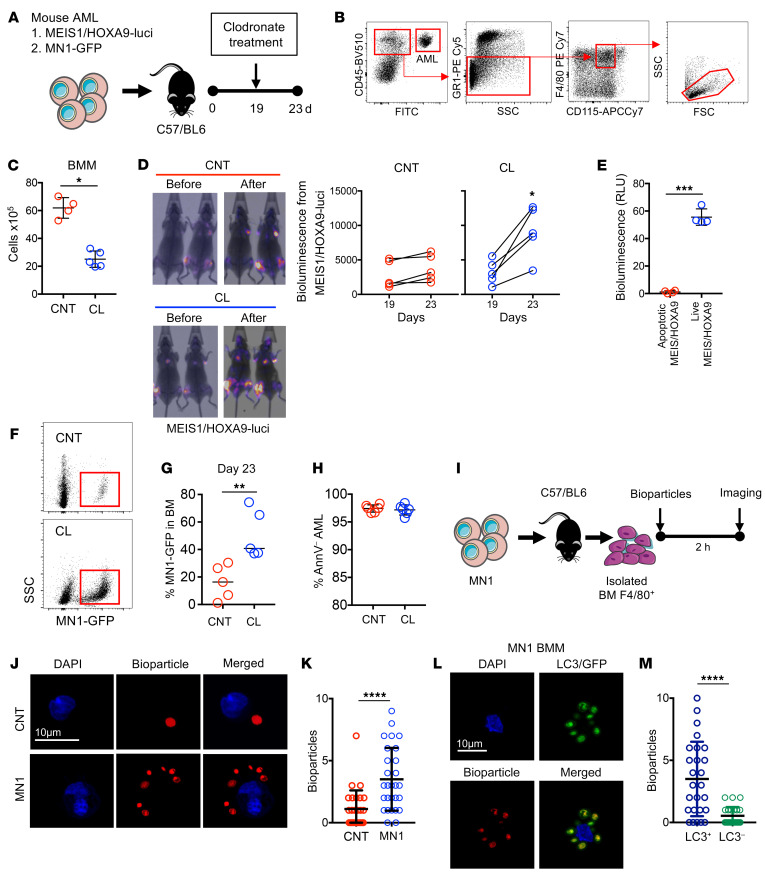
Depletion of phagocytes increases AML tumor burden. (**A**) MEIS/HOXA9 cells transduced with rLV.EF1.mCherry-Mito9 lentivirus (MEIS/HOXA9-luci) or MN1-GFP cells (1 × 10^6^) were injected into busulfan-treated C57/BL6 mice. Animals were treated with either CNTs or CLs on day 19 after injection and sacrificed on day 23. (**B**) Representative flow plot and gating strategy for BMMs (CD45^+^GR1^–^CD115^lo/int^F4/80^+^). FSC, forward scatter; SSC, side scatter. (**C**) Flow cytometric analysis of the number of BMMs (*n =* 5 mice). (**D**) In vivo imaging analysis of animals engrafted with MEIS/HOXA9-luci on days 19 and 23, representing before and after CL and CNT treatment. Graphs show bioluminescence analysis of the CL and CNT treatment groups (*n =* 5 mice). (**E**) Bioluminescence analysis of apoptotic and live MEIS/HOXA9-luci cells. (**F**) Representative flow plots for MN1-GFP engraftment in the CL and CNT treatment groups. BM was extracted and analyzed for (**G**) MN1-GFP engraftment and (**H**) annexin V staining (AnnV^+^ AML) of MN1-GFP cells (*n =* 5 mice). (**I**) MN1- or vehicle-treated (PBS) cells (1 × 10^6^) were injected into busulfan-treated C57/BL6 mice, and BM was harvested 14 days later. BMM F4/80^+^ cells were isolated via magnetic separation and incubated with zymosan A bioparticles for 2 hours, followed by imaging via fluorescence microscopy. (**J** and **K**) The number of bioparticles (red) per macrophage was counted for control and MN1-associated BMMs via microscopy (*n =* 25 BMMs). Scale bar: 10 μm. (**L** and **M**) The number of bioparticles (red) and LC3 (green) per MN1-associated BMM was counted and compared with the number of bioparticles without LC3 (*n =* 25 BMMs). Scale bar: 10 μm. Data indicate the mean ± SD *P < 0.05, **P < 0.01, ***P < 0.001, and ****P < 0.0001, by 2-tailed Mann-Whitney *U* test.

To establish the role played by phagocytes in AML, we used clodronate liposomes (CLs) to deplete phagocytic macrophages in a series of experiments using 2 AML in vivo models: myeloid ecotropic viral integration site 1/homeobox A9–driven (MEIS1/HOXA9-driven) AML (36), containing a GFP and a luciferase reporter, and meningioma 1–driven (MN1-driven) AML (37), containing a GFP reporter, both of which allowed for detection using in vivo imaging as well as measurement of tumor burden by the presence of GFP^+^ cells (Figure 1A). We used flow cytometry to determine the frequency of BMMs, as previously described (ref. 38 and Figure 1B). Figure 1C shows successful BMM depletion in response to CL treatment compared with results in control liposome–treated (CNT-treated) animals. In vivo imaging of MEIS1/HOXA9-engrafted cells showed that animals depleted of phagocytic macrophages by CL treatment had increased AML tumor burden compared with those treated with CNT, 23 days after the MEIS1/HOXA9 injections (Figure 1D). No bioluminescence signal was detected in apoptotic MEIS1/HOXA9 cells (Figure 1E). Using a second model of mouse AML (MN1-GFP), we confirmed the observation that CL treatment resulted in increased AML tumor burden in the BM compared with that seen in CNT-treated animals (Figure 1, F–H, and Supplemental Figure 1, A and B; supplemental material available online with this article; https://doi.org/10.1172/JCI153157DS1). To determine whether AML-associated BMMs exhibit increased phagocytosis compared with control BMMs, we isolated BMMs (F4/80^+^ cells) from WT and MN1-engrafted animals and cultured them with zymosan A bioparticles (Figure 1I). We found that BMMs from AML-engrafted animals had increased phagocytosis of the bioparticles compared with BMMs from WT animals (Figure 1, J and K). To determine whether phagocytosis involved LC3, we examined LC3 recruitment to phagosomes in BMMs from MN1-engrafted animals, after incubation with zymosan A bioparticles. Figure 1, L and M, shows that phagosomes generated in BMMs from MN1-engrafted animals recruited LC3.

### AML disease progression is accelerated in LAP-deficient animals.

**Figure 2:**
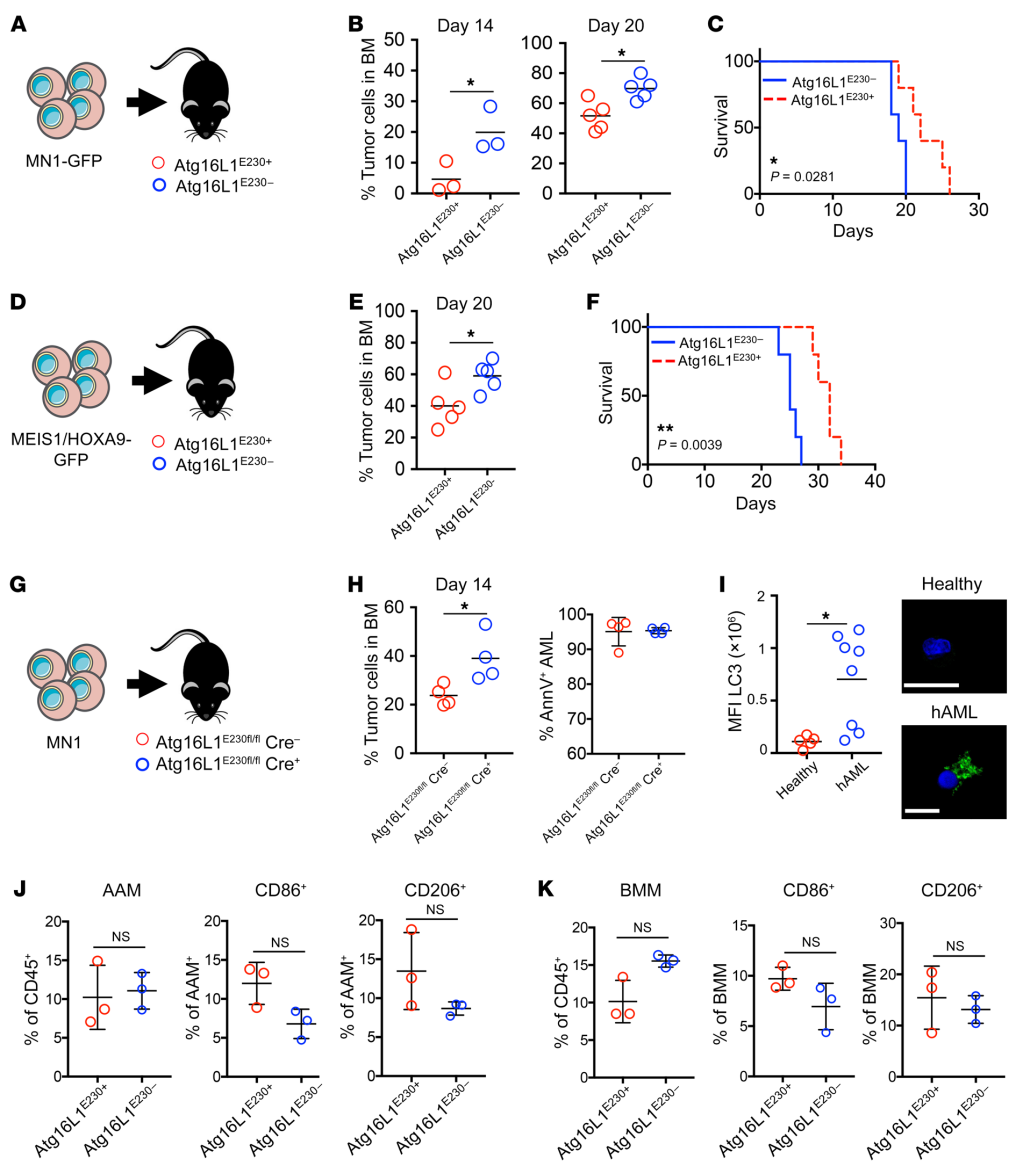
AML disease progression is accelerated in LAP-deficient animals. (**A**) MN1 cells (1 × 10^6^) were injected into busulfan-treated Atg16L1^E230+^ and Atg16L1^E230–^ mice. (**B**) BM was extracted and cells were analyzed by flow cytometry for engraftment on days 14 and 20 (*n =* 5 mice). (**C**) Kaplan-Meier curve showing the survival of Atg16L1^E230+^ and Atg16L1^E230–^ mice after injection (*n =* 5 mice). (**D**) MEIS/HOXA9 cells (1 × 10^6^) were injected into busulfan-treated Atg16L1^E230+^ and Atg16L1^E230–^ mice. (**E**) BM was extracted and cells were analyzed by flow cytometry for engraftment on day 20 (*n =* 5 mice). (**F**) Kaplan-Meier curve showing the survival of Atg16L1^E230+^ and Atg16L1^E230–^ mice after injection (*n =* 5 mice). (**G** and **H**) MN1 cells (1 × 10^6^) were injected into busulfan-treated Atg16L1^E230fl/fl^ Cre^+^ and Atg16L1^E230fl/fl^ Cre^–^ mice. BM was extracted and cells were analyzed by flow cytometry for engraftment on day 14 (*n =* 4 mice). (**I**) CD14^+^ cells were isolated from blood samples from patients with AML and controls. Cells were then fixed, permeabilized, and stained for LC3 and analyzed by microscopy (*n =* 5 healthy controls; *n =* 8 patients with AML). Scale bars: 10 µm. (**J**) MN1 cells (1 × 10^6^) or vehicle-treated (PBS) cells were injected into busulfan-treated Atg16L1^E230+^ and Atg16L1^E230–^ mice, and BM was harvested 14 days later. The percentage of AAMs (CD45^+^Lys6G^–^CD11b^+^) in the BM as well as the percentage of CD86^+^ and CD206^+^ AAMs were analyzed by flow cytometry (*n =* 3 mice). (**K**) The percentage of BMMs (CD45^+^GR1^–^CD115^lo/int^F4/80^+^) in the BM as well as the percentage of CD86^+^ and CD206^+^ BMM cells were analyzed via flow cytometry (*n =* 3 mice). Data indicate the mean ± SD. *P < 0.05 and **P < 0.01, by Mann-Whitney *U* test.

The recruitment of LC3 to phagosomes suggests a role for LAP during phagocytosis by BMMs. To understand the role of LAP in the tumor microenvironment of AML, LAP^–/–^ mice lacking the linker and WD domains of *Atg16L1* (Supplemental Figure 2 and ref. 39), which are required for LAP, but not canonical, autophagy (Atg16L1^E230–^), were injected with MN1 or MEIS1/HOXA9 cells. We then monitored the animals for tumor growth (Figure 2A). We observed that AML disease burden (by measuring GFP^+^ cells in the BM) was accelerated in Atg16L1^E230–^ mice compared with WT controls (Atg16L1^E230+^) as determined on days 14 and 20 after injection with MN1-driven AML (Figure 2B). As expected, increased tumor growth in the LAP-deficient animals was associated with decreased survival (Figure 2C). We observed similar results (by measuring GFP^+^ cells in the BM) using our MEIS/HOXA9-driven AML model. There were more AML blasts in the BM of Atg16L1^E230–^ animals, as well as decreased survival of Atg16L1^E230–^ mice compared Atg16L1^E230+^ animals (Figure 2, D–F). To engraft MN1, we conditioned the mice with a sublethal dose of busulfan. To determine whether busulfan changes BM cellularity, we treated Atg16L1^E230–^ and Atg16L1^E230+^ mice with busulfan alone and compared BM cellularity with that of control mice of each genotype. We observed no significant changes in BM cellularity (Supplemental Figure 3). As a second approach, we performed conditional targeting in macrophages and granulocytes using lysozyme M-Cre-lox recombination to generate Atg16L1^E230fl/fl^ Cre^+^ mice. MN1 cells were injected into Atg16L1^E230fl/fl^ Cre^–^ and Atg16L1^E230fl/fl^ Cre^+^ mice. We found that the AML tumor burden was increased in Atg16L1^E230fl/fl^ Cre^+^ mice compared with that in Atg16L1^E230fl/fl^ Cre^–^ mice (Figure 2, G and H, and Supplemental Figure 1C). To determine whether LAP is important in human AML, we examined LC3 density in isolated CD14^+^ cells. The data showed that LC3 density was higher in CD14^+^ cells from patients with AML than in controls (Figure 2I and Supplemental Table 1). These data suggest that LAP is an important process in reducing AML disease burden.

Next, we assessed the macrophage phenotype within the BM of AML-engrafted mice. AML TAMs have been characterized as CD45^+^Lys6G^–^CD11b^+^ macrophages and are called AML-associated macrophages (AAMs) (28), whereas tissue-resident BMMs express CD45^+^GR1^–^F4/80^+^CD115^int^ (Supplemental Figure 4A and 38). C57BL/6 mice were injected with MN1 cells, and BM was analyzed 14 days later. The percentage of AAMs in the CD45^+^ cell population were increased in animals engrafted with MN1 compared with controls. Furthermore, AAMs had decreased CD86 expression (an indicator of the M1 phenotype) compared with controls. We found no difference in expression of the M2 marker CD206 between the treatment groups (Supplemental Figure 4B). We found that BMMs were also increased in mice injected with MN1 cells, with an increase in CD86 and CD206 expression compared with that in controls (Supplemental Figure 4B). Next, we examined AAM and BMM phenotypes in Atg16L1^E230–^ mice compared with Atg16L1^E230+^ mice engrafted with MN1. We found no observable differences between the immunophenotypes of AAMs or BMMs when MN1 cells were injected into Atg16L1^E230–^ mice compared with that seen in Atg16L1^E230+^ mice (Figure 2, J and K). This shows that, although AML changed the phenotype and number of macrophages in the BM, deficiency in LAP did not alter this response.

### BMM LAP mediates clearance of AML apoptotic cells.

**Figure 3:**
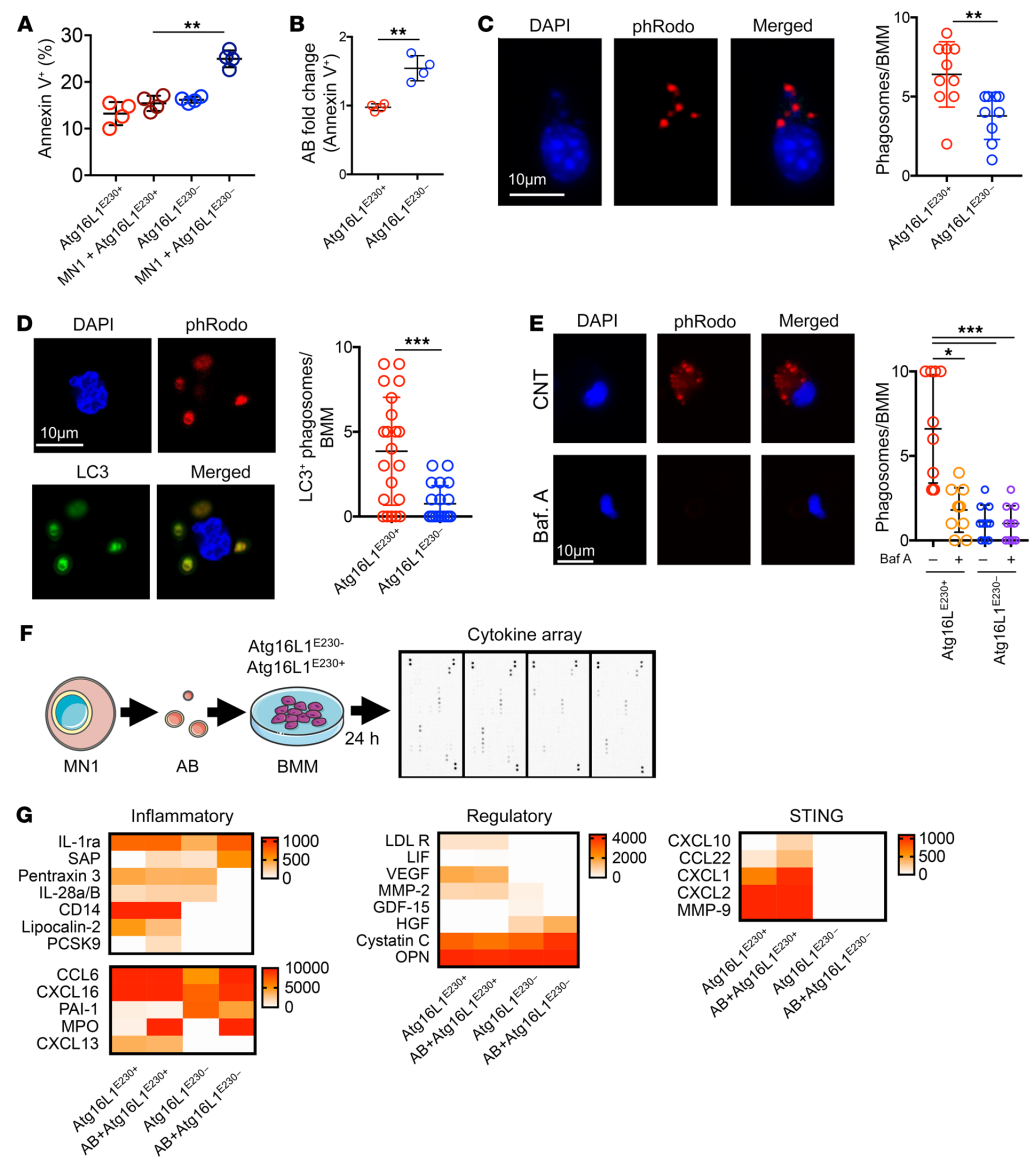
LAP in BMMs mediates AML AC clearance and activates the STING pathway. (**A**) MN1- or vehicle-treated (PBS) cells (1 × 10^6^) were injected into busulfan-treated Atg16L1^E230+^ and Atg16L1^E230–^ mice. BM was harvested 14 days after injection, and the percentage of annexin V^+^ cells in the BM was analyzed by flow cytometry (*n =* 5 mice). (**B**) Percentage of annexin V^+^ debris found in Atg16L1^E230+^ and Atg16L1^E230–^ mice engrafted with MN1 cells as determined by flow cytometry (*n =* 5 mice). (**C**) MN1-derived ABs were isolated via centrifugation and cultured with Atg16L1^E230+^ or Atg16L1^E230–^ BMMs for 3 hours. Representative fluorescence microscopy images of BMMs cultured with pHrodo-labeled ABs. The number of phagosomes per Atg16L1^E230+^ or Atg16L1^E230–^ BMM was counted (*n =* 10). Scale bar: 10 μm. (**D**) MN1-derived ABs were isolated via centrifugation and cultured with Atg16L1^E230+^ or Atg16L1^E230–^ BMMs for 3 hours. Representative fluorescence microscopy images show BMMs cultured with pHrodo-labeled ABs and stained for LC3. The number of phagosomes per Atg16L1^E230+^ or Atg16L1^E230–^ BMM was counted (*n =* 25). Scale bar: 10 μm. (**E**) MN1-derived ABs were isolated via centrifugation and cultured with Atg16L1^E230+^ or Atg16L1^E230–^ BMMs for 3 hours with or without bafilomycin (Baf A; 1 μM). Shown are representative fluorescence microscopy images of BMMs cultured with pHrodo-labeled ABs. Scale bar: 10 μm. Plot shows the quantification of phagosomes per Atg16L1^E230+^ or Atg16L1^E230–^ BMM (*n =* 10). (**F**) MN1 ABs were isolated and cultured with Atg16L1^E230+^ or Atg16L1^E230–^ BMMs for 24 hours. The supernatant was removed and centrifuged at 10,000 rpm for 10 minutes to remove any debris before performing a cytokine array. (**G**) Quantification of cytokine array results segmented into inflammatory, regulatory, and STING-related cytokines. Data indicate the mean ± SD. *P < 0.05, **P < 0.01, and ***P < 0.001, by Mann-Whitney *U* test (**B**–**D**) or Kruskal-Wallis test (**A** and **E**).

As the recognition of apoptotic tumor cells has previously been reported to promote antitumor immunity (40–42), we hypothesized that a link exists between reduced clearance of AML apoptotic cells (ACs) and apoptotic bodies (ABs) with increased leukemia progression. We measured ACs in LAP-deficient animals with AML and compared the results with ACs in WT control animals with AML. The gating strategy for this is shown in Supplemental Figure 1A. We found that the percentage of annexin V^+^ staining was higher in LAP-deficient Atg16L1^E230–^ animals with AML than in WT Atg16L1^E230+^ animals with AML (Figure 3A). Furthermore, Atg16L1^E230–^ AML-engrafted animals had increased annexin V^+^ debris compared with WT Atg16L1^E230+^ controls with AML, indicating a defect in the clearance of ABs and ACs in the BM of the Atg16L1^E230–^ animals (Figure 3B). To determine whether LAP is important in the clearance of AML ABs, we induced apoptosis in MN1 cells in vitro and isolated the ABs (Supplemental Figure 5). Isolated ABs were labeled with pHrodo to create pHrodo-ABs, which, when phagocytosed, cause the ABs to fluoresce. We cultured pHrodo-ABs with BMMs from Atg16L1^E230+^ and Atg16L1^E230–^ mice and found that Atg16L1^E230+^ BMMs had increased phagocytosis of ABs compared with Atg16L1^E230–^ BMMs (Figure 3C). Next, we assessed whether LC3-localized phagosomes occur more frequently in Atg16L1^E230+^ BMMs than in Atg16L1^E230–^ BMMs. Costaining the pHrodo-AB phagosome with anti–LC3-GFP showed that Atg16L1^E230+^ BMMs had significantly more LC3-localized phagosomes than did Atg16L1^E230–^ BMMs (Figure 3D). To confirm that BMMs from Atg16L1^E230+^ mice deliver ABs to the lysosomes and that this does not happen in Atg16L1^E230–^ BMMs, we used the lysosomal inhibitor bafilomycin A. Figure 3E shows that bafilomycin A inhibited the delivery of ABs to lysosomes in Atg16L1^E230+^ mice and that this did not happen in Atg16L1^E230– ^BMMs. These data demonstrate that LAP enhanced the clearance of apoptotic AML cells and debris in the BM.

### LAP induces STING in BMMs, which suppresses AML growth.

**Figure 4:**
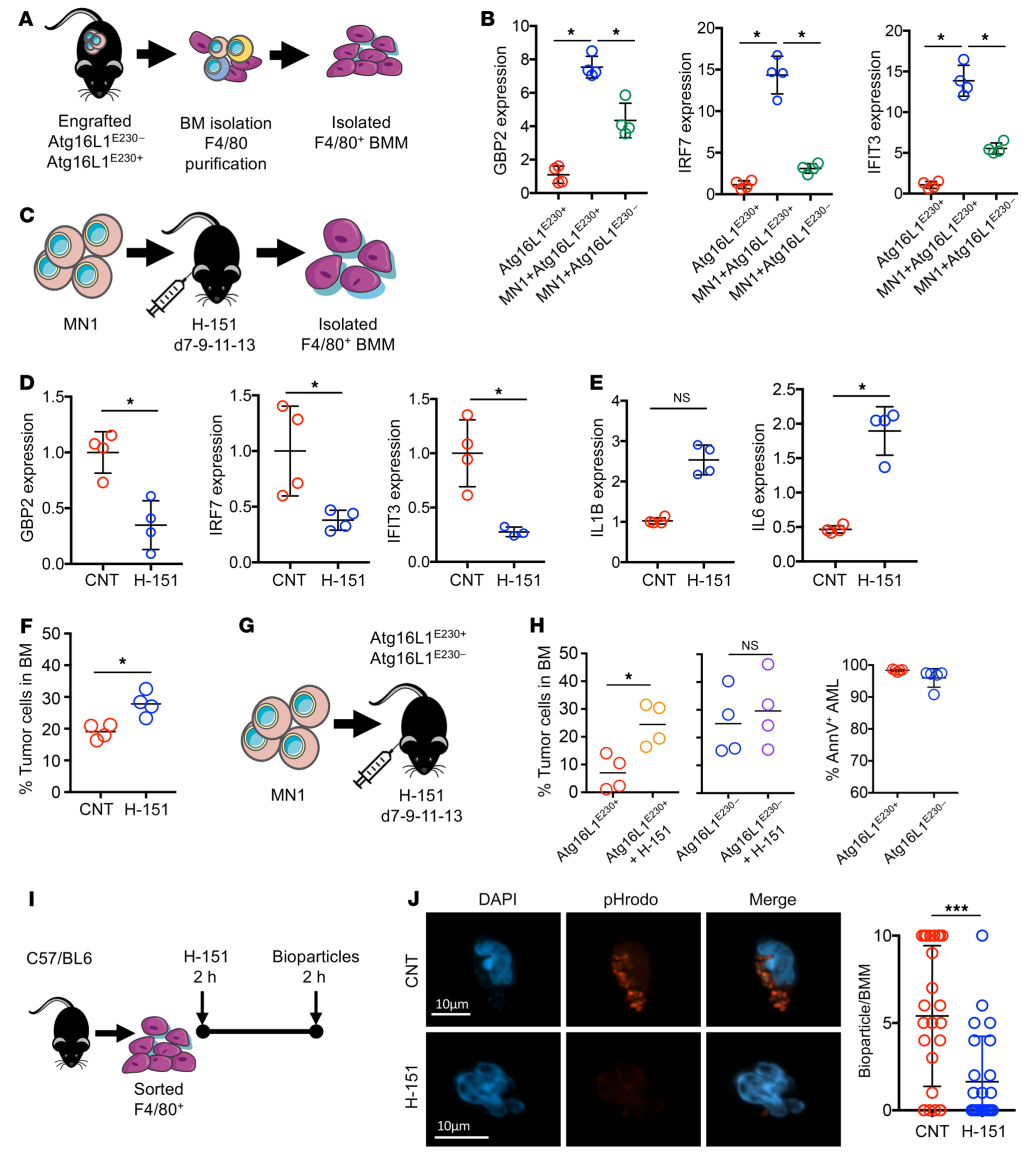
LAP activates STING in BMMs, reducing AML engraftment. (**A**) MN1-treated cells or vehicle-treated (PBS) cells (1 × 10^6^) were injected into busulfan-treated Atg16L1^E230+^ and Atg16L1^E230–^ mice. BMM F4/80^+^ cells were isolated via magnetic separation, and RNA was extracted for qPCR analysis. (**B**) Relative gene expression untreated, engrafted Atg16L1^E230+^, and engrafted Atg16L1^E230–^ isolated BMMs (*n =* 4 mice). (**C**) MN1 cells (1 × 10^6^) were injected into busulfan-treated C57/BL6 mice. On days 7, 9, 11, and 13 after injection, mice were injected i.p. with 200 μL H-151 (750 nmol) or vehicle and sacrificed 14 days later. BMMs were sorted and RNA was extracted for analysis by qPCR. (**D**) Relative gene expression in AAMs from animals engrafted with MN1 cells or MN1 cells treated with H-151. (**E**) Relative expression of *IL1B* and *IL6* genes (*n =* 4 mice). (**F**) BM was extracted, and cells were analyzed by flow cytometry for engraftment on day 14 (*n =* 4 mice). (**G** and **H**) MN1 cells (1 × 10^6^) were injected into busulfan-treated Atg16L1^E230+^ and Atg16L1^E230–^ mice. On days 7, 9, 11, and 13 after injection, mice were injected i.p. with 200 μL H-151 (750 nmol) or vehicle and sacrificed 14 days later. BM was extracted and cells were analyzed by flow cytometry for engraftment on day 14 (*n =* 4 mice). (**I**) Schematic diagram of the experimental design. F4/80^+^ BMMs from C57/BL6 mice were sorted and cultured with H-151 (10 μM) or vehicle for 2 hours. BMMs were then incubated with pHrodo *E. coli* bioparticles for 2 hours. (**J**) Representative microscopy images. Scale bars: 10 μm. Plot shows the quantification of bioparticles (red) per BMM in control and H-151–treated BMMs (*n =* 25 BMMs). Data indicate the mean ± SD. *P < 0.05 and ***P < 0.001, by Mann-Whitney *U* test (**D**–**F**, **H**, and **J**) and Kruskal-Wallis test (**B**).

To investigate the impact of LAP in regulating AML progression, we induced apoptosis in MN1 cells and isolated the ABs and cocultured them with BMMs from Atg16L1^E230+^ and Atg16L1^E230–^ animals for 24 hours. We assayed the cell supernatant for cytokine profile using Proteome Profiler Mouse XL Cytokine Arrays (Figure 3F and Supplemental Figure 6). Pathway analysis before and after AB treatment revealed that cytokines and chemokines related to STING activation were present in the supernatant from Atg16L1^E230+^ BMMs, but absent in that from Atg16L1^E230–^ BMMs (Figure 3G). BMMs isolated from Atg16L1^E230+^ and Atg16L1^E230–^ animals engrafted with AML were therefore examined for STING activation by measuring *Gbp2*, *Irf7*, and *Ifit3* gene expression (Figure 4A and refs. 35, 43). BMM from AML-engrafted Atg16L1^E230+^ animals showed pronounced activation of STING, indicated by increased expression of *Gbp2*, *Irf7*, and *Ifit3* compared with BMMs from AML-engrafted Atg16L1^E230–^ animals (Figure 4B). To confirm that the increased cytokine expression resulted from activation of STING, we treated C57/BL6 mice engrafted with MN1 cells over 7 days with the STING inhibitor H-151. The animals were then sacrificed, the BM was isolated, and the BMMs were FACS-purified and analyzed for *Gbp2*, *Irf7*, and *Ifit3* gene expression (Figure 4C). When compared with control animals, BMMs from H-151–treated animals had decreased expression of the STING markers *Gbp2*, *Irf7*, and *Ifit3* (Figure 4D), but increased expression of the proinflammatory cytokines *Il1b* and *Il6* (Figure 4E). Furthermore, animals treated with H-151 had an increase in AML tumor burden (Figure 4F). To confirm the role of LAP, we used the STING inhibitor H-151 in the Atg16L1^E230^ mouse model (Figure 4G). Figure 4H shows that the STING inhibitor enhanced the tumor burden in Atg16L1^E230+^ mice but not in Atg16L1^E230–^ mice.

In solid tumors, STING activation promotes recognition and killing of cancer cells via mechanisms that include both enhancement of cancer antigen presentation and regulation of CD8^+^ T cell trafficking and infiltration into tumors (44–46). To understand the antitumoral effects of STING in AML, we first looked at T cell migration into the BM and subsequent activation. Post-engraftment analysis showed that CD4^+^ cells were increased in MN1-engrafted animals compared with control animals, but we observed no changes in the percentage of CD8^+^ cells or their IFN-γ status (Supplemental Figure 7). Since we observed increased phagocytosis in AML-primed BMM compared with naive BMMs, we assessed the phagocytic capacity of F4/80^+^ BMM following STING inhibition by H-151. Figure 4, I and J, shows that inhibition of STING reduced phagocytosis in BMMs of pHrodo bioparticles compared with control BMMs. Therefore, unlike solid tumors, the antitumoral effect of BMM STING activation in AML functions by upregulating the phagocytotic potential.

### AML-derived ABs contain mitochondria that are processed by BMMs.

**Figure 5:**
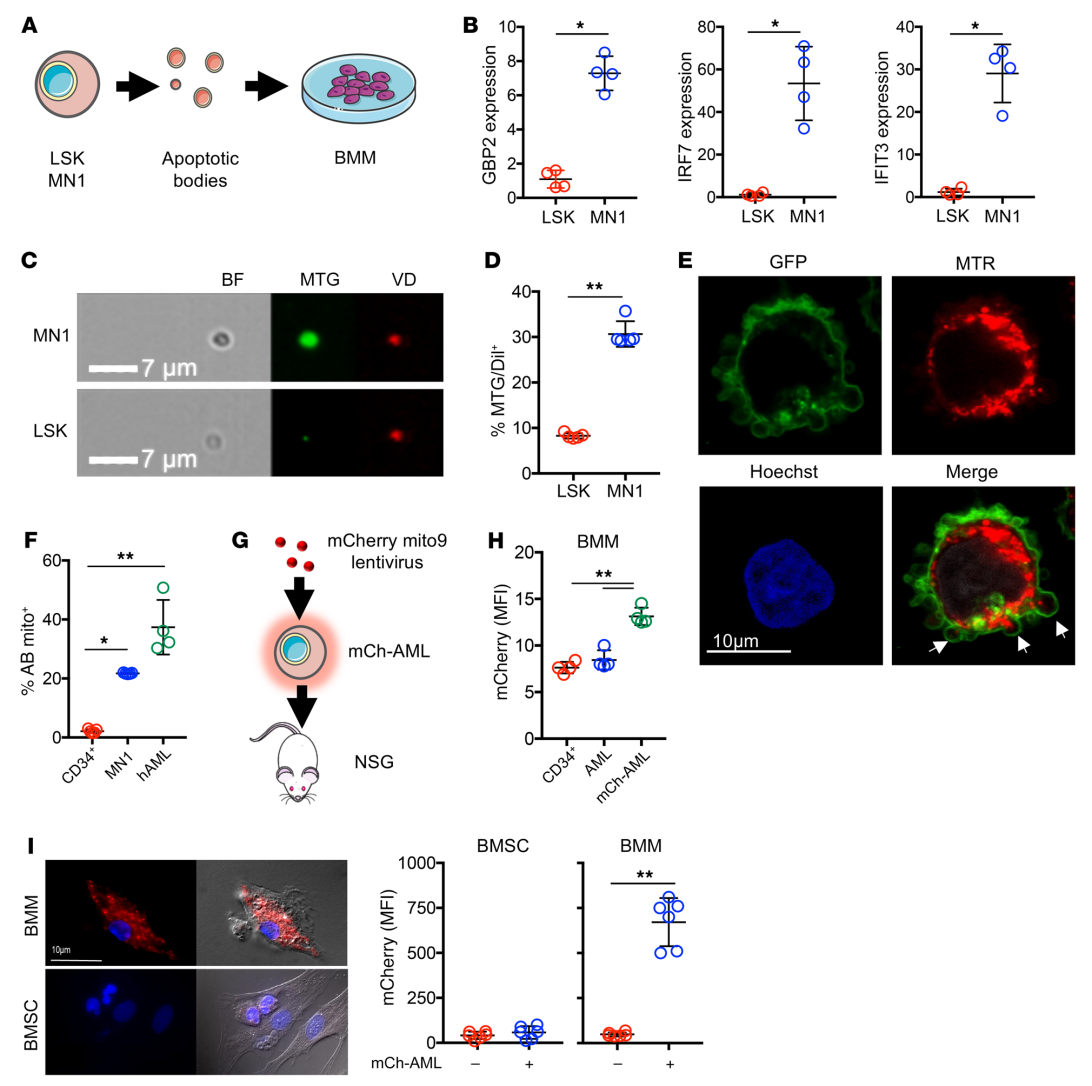
AML-derived ABs contain mitochondria that are processed by BMMs. (**A**) ABs were isolated from MN1 and nonmalignant LSK cells and cultured with BMMs from C57/BL6 mice for 24 hours, and RNA was extracted for qPCR analysis. (**B**) Relative gene expression of *Gbp2*, *Irf7*, and *Ifit3* in BMMs cultured with MN1 and LSK ABs (*n =* 5). (**C**) Representative images of nonmalignant LSK and MN1 cells stained with MitoTracker Green (MTG) or VybrantDil (VD). ABs were isolated and analyzed via image flow cytometry. Scale bar: 7 μm. (**D**) Percentage of ABs from LSK and MN1 cells that were positive for MitoTracker Green and VybrantDil (*n =* 5). (**E**) Representative confocal microscopy images of human AML cells that were transduced with a GFP membrane virus and stained with MitoTracker Red (MTR) and Hoechst. Arrows indicate blebs containing mitochondria. Scale bar: 10 μm. (**F**) Nonmalignant CD34, MN1, and human AML cells were stained with MitoTracker Red before isolating the ABs and analyzing them via flow cytometry for the percentage of ABs containing MitoTracker Red (mito^+^) (*n =* 5). (**G**) Schematic diagram of the experimental design. Primary AML cells were transduced with rLV.EF1.mCherry-Mito9 lentivirus (mCh-AML) and injected into NSG mice and left for 35 days (*n =* 3). (**H**) BM was extracted and BMMs were analyzed by flow cytometry for mCherry fluorescence (MFI). (**I**) mCh-AML cells were cocultured with BMSCs and BMMs and analyzed by microscopy for mitochondria uptake, as determined by mCherry MFI (*n =* 25). Scale bar: 10 μm. Data indicate the mean ± SD. *P < 0.05 and **P < 0.01, by Mann-Whitney *U* test (**B**, **D**, and **I**) and Kruskal-Wallis test (**F** and **H**).

As self-DNA (nuclear and/or mitochondrial) has been shown to stimulate STING in autoinflammatory and malignant disease (47–50), and both AML growth and chemotherapy-induced DNA damage dysregulate the BM apoptotic response (51, 52), we hypothesized that AML-specific BMM STING activation is mediated by local tumor cell apoptosis and cellular debris. To address this question, we isolated AML-derived ABs from MN1 cells and nonmalignant lineage-negative, SCA-positive, KIT-positive (LSK) cells (as controls, a surrogate of HSPCs). We cultured these cells ex vivo with BMMs for 24 hours before analysis of STING-induced gene expression by quantitative PCR (qPCR) (Figure 5A). MN1-derived ABs caused upregulation in BMM STING–related genes when compared with control LSK-derived ABs (Figure 5B). Activation of STING has been shown to occur in response to mitochondrial damage-associated molecular patterns (mtDAMPs), including mitochondrial DNA (mtDNA) (53, 54). Previously, we and others have shown that AML cells contain a higher mitochondrial mass than do nonmalignant LSK cells (Supplemental Figure 8 and refs. 55, 56). Therefore, we assessed whether ABs from AML contained mitochondria. We first measured mitochondrial content in AML-derived ABs and LSK ABs using MitoTracker Green and VybrantDil membrane stain and analyzed ABs via image flow cytometry. We observed that MN1-derived ABs had increased mitochondrial content compared with LSK-derived ABs (Figure 5C). Additionally, we looked at mitochondrial association with the ABs and found that MitoTracker Green and VybrantDil association was increased in MN1-derived ABs compared with LSK-derived ABs (Figure 5D). Furthermore, by confocal microscopy, we directly visualized cell membrane blebs containing mitochondria (Figure 5E). These data demonstrate that the AML-derived ABs contained mitochondria.

Second, to understand whether this process is unique to the mouse models used or translates to human disease, we stained 5 separate human AML BM samples, MN1, and nonmalignant human CD34^+^ cells with MitoTracker Red and isolated the ABs. Human AML– and MN1-derived ABs had significantly more MitoTracker Red staining than did nonmalignant human CD34^+^–derived ABs (Figure 5F). To determine whether human AML mitochondria are processed by BMMs in vivo, we engrafted nonobese diabetic (NOD) SCID Il2rg-knockout (NOD.Cg.Prkd^scid^IL2rg^tm1Wji^/SzJ) (NSG) mice with human AML cells (Supplemental Figure 9, A and B) or human AML cells transduced with mCherry mito9 lentivirus (mCh-AML) to visualize mitochondria (Figure 5G). On day 35 after tumor cell injection (prior to the disease-associated terminal end point), the animals were sacrificed, and analysis of the BMMs from mCh-AML–engrafted animals showed increased mCherry fluorescence compared with BMMs from control animals transplanted with nonmalignant CD34^+^ cells (Figure 5H). To confirm this BMM-mediated phenotype, we cocultured mCh-AML cells ex vivo with either BMMs or BM stromal cells (BMSCs) and analyzed the macrophages and stromal cells for mCherry uptake via fluorescence microscopy. We found that BMMs had increased mCherry fluorescence compared with BMSCs (Figure 5I). These data show that AML-derived ABs containing mitochondria were phagocytosed by BMM.

### AML-derived ABs containing mtDNA activate STING in BMMs via a LAP-dependent mechanism.

**Figure 6:**
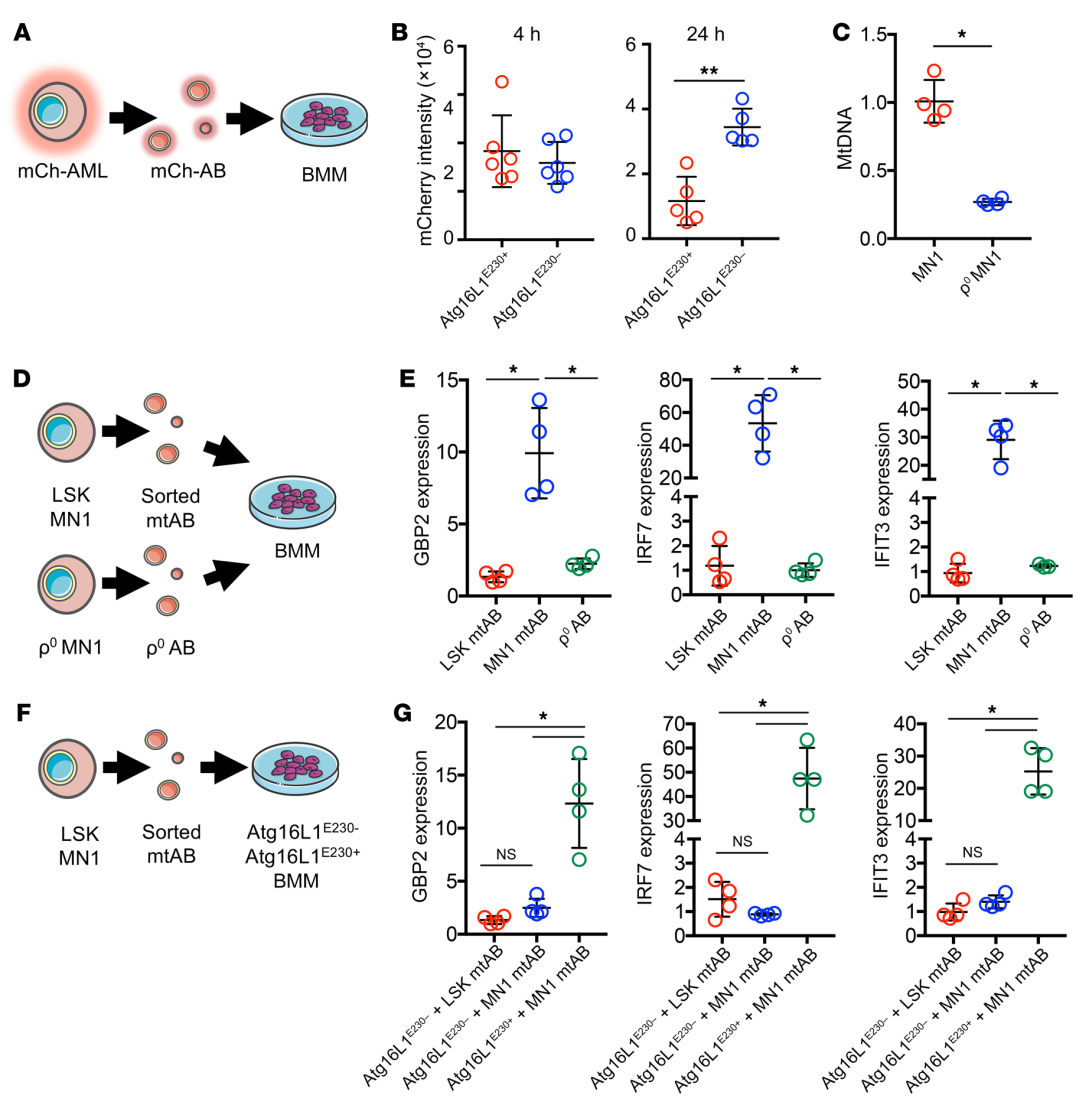
AML-derived ABs containing mtDNA activate STING in BMMs via a LAP-dependent mechanism. (**A**) Primary AML cells were transduced with rLV.EF1.mCherry-Mito9 lentivirus (mCh-AML), and the ABs were isolated (mCh-AB). mCh-ABs were cultured with BMMs from Atg16L1^E230+^ and Atg16L1^E230–^ mice for 24 hours. (**B**) mCherry intensity between Atg16L1^E230+^ and Atg16L1^E230–^ BMMs was analyzed at 4 and 24 hours by confocal microscopy (*n =* 5). (**C**) Relative mtDNA levels of MN1 cells and ρ^0^-generated MN1 cells normalized to DNA levels using TaqMan PCR and Tert and ND3 probes (*n =* 5). (**D**). Nonmalignant LSK cells and MN1 cells were stained for MitoTracker Red, and the ABs were isolated before being sorted on the basis of a positive MitoTracker Red signal. ρ^0^ MN1 cell ABs were also isolated. The ABs were cultured for 24 hours with BMMs from C57/BL6 mice, and RNA was extracted for qPCR analysis. (**E**) Relative gene expression of *Gbp2*, *Irf7*, and *Ifit3* in BMMs cultured with sorted mitochondria containing ABs from MN1, LSK, and ρ^0^ MN1 cells AB (*n =* 4). (**F**) Nonmalignant LSK and MN1 cells were stained for MitoTracker Red, and the ABs were isolated before sorting on the basis of a positive MitoTracker Red signal. The ABs were cultured for 24 hours with BMMs from Atg16L1^E230–^ and Atg16L1^E230+^ mice, and RNA was extracted for qPCR analysis. (**G**) Relative gene expression of *Gbp2*, *Irf7*, and *Ifit3* in Atg16L1^E230–^ and Atg16L1^E230+^ BMMs cultured with sorted mitochondria containing ABs from MN1 and LSK cells (*n =* 5). Data indicate the mean ± SD. *P < 0.05 and **P < 0.01, by Mann-Whitney *U* test (**B** and **C**) and Kruskal-Wallis test (**E** and **G**).

Next, as we observed that AML-derived ABs induced STING regulated gene activation and that mitochondria containing AML-derived ABs were processed by BMMs, we investigated whether LAP is required for phagocytosis of ABs containing mitochondria. We first isolated ABs from mCh-AML cells (mCh-AB) (Supplemental Figure 10) and cultured them ex vivo with BMMs from Atg16L1^E230+^ and Atg16L1^E230–^ animals for 24 hours (Figure 6A). After 4 hours, both Atg16L1^E230+^ and Atg16L1^E230–^ BMMs showed an increase in mCherry uptake. This was reduced after 24 hours in the Atg16L1^E230+^ BMMs, but not the Atg16L1^E230–^ BMMs, suggesting that Atg16L1^E230–^ BMMs took up the mCh-AB but were unable to deliver them to lysosomes for degradation (Figure 6B). Since mtDNA activates STING, we generated MN1 cells depleted of mtDNA (ρ^0^ MN1) by long-term culturing in ethidium bromide and 2′,3′-dideoxycytidine (Figure 6C). We isolated MN1 ρ^0^–derived ABs, as well as FACS-purified mitochondria containing ABs from MN1 and LSK cells, and cocultured the ABs with BMMs from C57/BL6 mice for 24 hours before performing qPCR to measure gene expression (Figure 6D). Although we detected an increase in the expression of STING-related genes in BMMs treated with MN1 mitochondria containing ABs, there was no increase in STING-related genes from BMMs cocultured with MN1 ρ^0^ AB (Figure 6E). To determine whether this response was LAP dependent, we performed FACS to isolate mitochondria containing ABs from MN1 and nonmalignant LSK cells (LSK was used as a control that makes ABs with low levels of mitochondria; see Figure 5D) and incubated these cells with Atg16L1^E230–^ or Atg16L1^E230+^ BMMs for 24 hours (Figure 6F). We then analyzed the BMMs for gene expression related to STING. Although we detected an increase in STING-related gene expression in Atg16L1^E230+^ BMMs treated with mtABs from MN1 cells, there were no such increases in the Atg16L1^E230–^ BMMs (Figure 6G). Together, these data show that LAP was required in BMMs to process AML-derived ABs containing mtDAMPs and resulted in the activation of STING.

## Discussion

The presence of TAMs is generally related to a poorer prognosis in patients with solid tumors (57–62). In contrast, we observed that in in vivo models of AML, generalized BMM depletion accelerated AML growth. This occurred because BMM phagocytosis of apoptotic cellular debris in the BM microenvironment resulted in the suppression of AML growth. Specifically, we found that phagocytosis of mtDAMPs induced STING activation in the BMMs, conferring an antitumoral phenotype. The activation of STING resulted in increased phagocytotic capacity of BMMs and inhibited AML progression, independent of T cell activation.

Our data describing the role of macrophages in the progression of AML appeared at first look contradictory. In some contexts, macrophages in the tumor microenvironment have been found to promote AML progression. Specifically, displacement of resident macrophages or invasion of tumor-supporting macrophages has been shown to correlate with low survival in patients with AML (29). Additionally, macrophages have the ability to protect AML cells from apoptosis (28). Furthermore, the immunosuppressive environment created by leukemic cells alters BMMs to reduce their phagocytic capacity and avoidance of immune regulation (63). Contrary to these studies, we and others have shown that depletion of macrophages using CLs increases AML engraftment (64). Moreover, we found that LAP in macrophages negatively regulated AML progression by altering the phagocytic potential of macrophages to promote increased phagocytic clearance. Furthermore, our results show that deficiencies of LAP in macrophages led to accumulation of apoptotic AML debris, which resulted in a tumor-supporting environment. The spectrum of apparently diverse roles of BMMs may be explained in part through observations leading to experimental subcategorization of macrophages by phenotypic polarization. Through this, distinct transcriptional programs are activated, resulting in defined patterns of cytokine response and protein expression profiles (20). A simplified conceptual framework has been developed to broadly divide macrophages into M1 and M2 subtypes (21), with “classically activated” macrophages (M1 macrophages) generally considered to be primed for pathogenic and antitumor responses (20) and “alternatively activated” macrophages (M2 macrophages) regarded as having immunoregulatory and protumoral functions (65). However, macrophage polarity likely represents a dynamic continuous spectrum of phenotypes, within which M1 and M2 polarity is regulated by microenvironmental cues (20, 23, 24). How these largely ex vitro–defined phenotypes relate to in vivo pathophysiology and explain the spectrum of macrophage functions remains to be explained.

Ecologically, tumors exhibit dynamic and synchronous cell death and proliferation. Apoptosis has previously been thought of as immunologically silent or even as a tolerogenic death modality (66–68). However, a growing body of evidence suggests an important role for uncleared apoptotic debris in stimulating immunologic responses in malignancies (40, 41, 69–71). Here, we identified LAP-mediated BMM clearance of AML apoptotic debris as an important regulator of AML disease progression. The suppression of AML by BMMs through a LAP-dependent mechanism is a phenotype not seen in solid tumors, to our knowledge. Studies using models of adenocarcinoma and melanoma models (35) report the recruitment of cytotoxic T cells into tumors in the context of LAP-deficient myeloid cells, resulting in an antitumoral response. In contrast, our results showed no increased activation or recruitment or of cytotoxic T cells into the leukemic BM in LAP-deficient animals.

In this study, we identified the importance of LAP in AML suppression using a mouse model that lacked the linker and WD domains of ATG16L1 from E230 in the amino acid sequence (Supplemental Figure 2 and refs. 39, 72). The conventional Atg16L1^E230–^ mouse model is not limited to the myeloid lineage, which may allow for nonmyeloid cells to be implicated in producing the results observed in this study. To overcome this limitation, we generated myeloid-specific Atg16L1^E230–^ mice, which when engrafted, showed an increased tumor burden compared with control mice. Other studies have used deletion of *RUBCN* (also known as *Rubicon*; *rubcn^–/–^* mice) (73, 74), which can be targeted to the myeloid cells. *RUBCN* is part of a complex upstream of ATG16L1 containing UVRAG, Beclin 1, and VPS34. *RUBCN* is essential for LAP, because it increases the class III PI3 kinase activity of VPS34 to generate phosphatidylinositol 3-phosphate [PI(3)P] on phagosomes to stabilize the NOX2 complex for the production of ROS and recruit the ATG5-ATG12:ATG16L1 complex to conjugate LC3 to the phagosome membrane (74). *Rubcn^−/–^* mouse models showed inefficient clearance of pathogens and ACs as well as elevated inflammation, leading to the development of autoimmune diseases such as systemic lupus erythematosus (SLE). Additionally, *rubcn^−/–^* mice were shown to induce a type I IFN response in tumor-infiltrating macrophages (35). *Rubcn^–/–^* mouse models are LAP deficient and could have been used to determine the role of LAP in AML progression.

We and others have shown that the mitochondrial content of AML cells is increased compared with nonmalignant hematopoietic and progenitor cells (55, 56). Unlike many tumors that rely on the Warburg effect for energy production, AML primarily uses oxidative phosphorylation (OXPHOS) for ATP production, hence the increased mitochondrial mass (75). The increased mitochondrial mass and rapid growth of the AML cells leads to the generation of dysfunctional mitochondria and ultimately to an increase in ROS as a by-product of OXPHOS (76). Dysfunctional mitochondria also initiate signal cascades for apoptosis (77–79). In tumor evolution, an increasing cell turnover rate slows tumor growth but accelerates the rate of evolution for both proliferation and migration (80). Increasing cell turnover also results in increased numbers of ACs. ACs generate membrane blebs that release from the cell in ABs and contain cellular components including mtDAMPs (54). mtDNA has been shown to activate STING via cyclic GMP-AMP synthase (cGAS) to cause downstream effects, such as a type I IFN response (53). A previous investigation of AML STING activation has shown that a AML type I IFN response is not triggered when compared with solid tumors, where STING activation is responsible for the maturation of DCs (81). Here, we have shown that mtDNA from AML-derived ABs was processed by macrophages in a LAP-dependent manner. Furthermore, we observed that inhibition of STING in BMMs led to decreased phagocytic potential, increased AML progression, and no changes in T cell activation. Accordingly, AML rapid cell proliferation was driven by ATP, primarily from OXPHOS (over glycolysis), but occurred with the metabolic cost of increased ROS, apoptosis, and dysfunctional mitochondria released into the BM microenvironment. These observations reveal tumor-specific vulnerabilities and present strategic opportunities when considering novel approaches to managing patients with AML. Clinically, shifting the balance between cell turnover and tumor growth may be possible through the regulation of processing of ROS and ACs in the BM microenvironment

Recently, CD47 has been identified as a “do-not-eat-me” signal, which is overexpressed in myeloid malignancies (30, 82, 83). Blockade of CD47 leads to engulfment of leukemic cells, with preclinical studies demonstrating antimalignant activity in AML and myelodysplastic syndrome (MDS) (32, 84). Subsequently, clinical studies have been initiated with CD47-targeting agents in both AML and MDS as monotherapy and in combination with chemotherapy (31). Others have shown that STING activation using 5,6-dimethylxanthenone-4-acetic acid (DMXAA) can significantly extend survival in in vivo models of AML (81). In our model, LAP-dependent activation of STING increased the phagocytosis of AML cells. In mouse melanoma cells and several other cell lines, the anthracycline drug doxorubicin induced activation of STING. Therefore, as anthracycline chemotherapy has long been the standard of care for younger patients with AML (85), these data combined led us to hypothesize that chemotherapy-induced STING activation in macrophages may enhance the anti-AML effects of CD47 inhibition.

These experimental data, set within the context of the existing literature, highlight the diversity of macrophage phenotypic function in AML. This diversity in function is dynamic and entirely dependent on the cell-, tissue-, treatment-, and disease-specific context induced by the tumor and its microenvironment. Accordingly, the appreciation of the relationship between proliferation and apoptosis, set within the broader chemotherapy context, will be vital when planning the drug sequence and timing of treatment strategies looking to harness the therapeutic potential of macrophages in the management of AML.

## Methods

### Animals.

Nonobese diabetic (NOD) SCID Il2rg-knockout (NOD.Cg.Prkd^scid^IL2rg^tm1Wji^/SzJ)(NSG) mice were purchased from The Jackson Laboratory. C57BL/6J mice were purchased from Charles River Laboratories. The generation of LAP-deficient mice (Atg16l^E230–^) has been previously described (39). Atg16L1^E230fl/fl^ Cre^+^ mice were generated by crossing mice containing the lysozyme M–Cre promoter with mice containing floxed sites flanking exon 2 of the *ATG16L1* gene. Animals were housed in a specific pathogen–free facility. Mice used in these experiments were 8–12 weeks of age and were of both sexes.

### Primary cells.

Nonmalignant and malignant hematopoietic cells were collected at the Norfolk and Norwich University Hospital. CD34^+^ HSCs were isolated via density-gradient centrifugation and CD34^+^ microbeads (Miltenyi Biotec). To engraft nonmalignant CD34^+^ cells into NSG mice, 3 doses of 25 mg/kg of busulfan were given on day –3, day –2 and day –1, followed by injection of nonmalignant CD34^+^ on day 0. Primary AML blasts were obtained from the BM of patients with AML (Table 1). AML cell isolation was performed by density-gradient centrifugation using Histopaque (MilliporeSigma). AML cells were cultured in DMEM containing 10% FBS plus 1% penicillin-streptomycin. To generate mCherry mitochondria in primary AML cells, cells were seeded at a density of 5 × 10^4^ in 500 μL DMEM supplemented with 10% FBS and transduced with 0.5 μL rLV.EF1.mCherry-Mito9 lentivirus (Clontech Takara Bio Europe). AML cells were cultured for 1 additional week to ensure that no residual lentivirus remained. Transduction was confirmed by the detection of mCherry fluorescence in AML cells using fluorescence microscopy. To engraft human AML cells into NSG mice, busulfan was not used; rather, AML cells were injected into the NSG mice without conditioning.

### BM isolation.

Isolation of BM was achieved by isolating the femur and tibia from each mouse. Each bone was cut centrally and placed into a 0.5 mL microcentrifuge tube containing a hole allowing the removal of the BM from the bone. This was placed in a 1.5 mL microcentrifuge and centrifuged for 6 seconds to allow collection of BM cells. The BM pellet from each mouse was collected and washed in MACS buffer (1× PBS, 0.5% BSA, 2 mM EDTA, filtered) before being passed through a 40 μm CellTrics filter (Sysmex). The BM was isolated from 1 tibia and 1 femur, and the cells were counted via an automated cell counter (Cellometer Auto T4, Nexcelom Bioscience). Absolute live cell numbers and AML cell numbers were calculated by multiplying the frequencies of cells by the total numbers of cells per tibia and femur.

### LSK cell isolation.

Mouse LSK cells were isolated from mouse BM using Lin^–^ microbeads followed by CD117 microbeads (Miltenyi Biotec) and then sorted for SCA1-APC (Miltenyi Biotech) on a SH800S Cell Sorter (Sony). The LSK cells were expanded in DMEM containing 10% FBS plus 1% penicillin-streptomycin supplemented with mouse stem cell factor (mSCF; 100 ng/mL), mouse IL-3 (mIL-3; 10 ng/mL), and human IL-6 (hIL-6; 10 ng/mL) (Peptrotech).

### BMMs.

Mouse BMMs were isolated from cultured mouse BM in RPMI-1640 (Gibco, Thermo Fisher Scientific) containing 20% FBS (Gibco, Thermo Fisher Scientific) plus 1% penicillin-streptomycin (Gibco, Thermo Fisher Scientific) supplemented with 20 ng/mL macrophage CSF (Peptrotech). Briefly, 1 × 10^7^ to 2 × 10^7^ BM cells were plated onto nontissue-cultured treated 10 cm plastic dishes, with fresh media added on day 3. On day 6 or day 7, cells were washed with 1× PBS, followed by addition of cold PBS, and cells were removed by scraping. After cell numbers were established, BMMs were plated in RPMI-1640 containing 20% FBS plus 1% penicillin-streptomycin for 24 hours before experimental use. For longer survival, BMMs were cultured in RPMI-1640 containing 20% FBS plus 1% penicillin-streptomycin supplemented with macrophage CSF (10 ng/mL). Mouse BMM markers were confirmed by flow cytometry for F4/80^+^ and GR1^–^ expression. F4/80^+^ cells from BM samples were isolated using positive selection with F4/80 microbeads (Miltenyi Biotec).

### BMSC isolation.

Mouse BSMCs were isolated from mouse BM by adherence to tissue culture plastic and then cultured in MEM containing 20% FBS plus 1% penicillin-streptomycin. Mouse BMSC markers were confirmed via flow cytometry for CD45^–^, CD31^–^, Ter119^–^, CD105^+^, and CD140a^+^ expression.

### MN1 and MEIS1/HOXA9 cells.

MN1 and MEIS1/HOXA9 cells were generated as previously described (86) and maintained in DMEM containing 10% FBS plus 1% penicillin-streptomycin supplemented with mSCF (100 ng/mL), mIL-3 (10 ng/mL), and hIL-6 (10 ng/mL) (Peptrotech). MEIS1/HOXA9 cells were infected with pCDH-luciferase-T2A-mCherry for in vivo imaging and were provided by Irmela Jeremias (Helmholtz Zentrum München, Munich, Germany; ref. 87). Transduced MEIS1/HOXA9 cells (MEIS1/HOXA9-luci) were sorted using mCherry fluorescence on a BD FACSMelody (BD Biosciences). Culturing of cells was carried out in 5% CO_2_ at 37°C. To engraft AML cells into WT and Atg16l^E230–^ mice, 2 doses of 25 mg/kg busulfan were i.p. injected on day –2 and day –1, followed by i.v. tail-vein injection of MN1 and MEIS1/HOXA9 cells on day 0.

### CL experiment.

C57/BL6 mice were treated i.p. with 25 mg/kg busulfan on day –2 and day –1 prior to tail-vein injections of 1 × 10^6^ MEIS/HOXA9-luci cells or MN1-GFP cells on day 0. Nineteen days after injection, the animals were imaged via in vivo bioluminescence imaging (Bruker) and then injected i.p. with either 150 μL CLs or CNTs (Stratech). Animals were imaged and sacrificed on day 23 after injection. Bioluminescence levels of MEIS/HOXA9-luci cells were analyzed using ImageJ (Fiji, NIH). Macrophage numbers (CD45^+^, GR1^–^, CD115^lo/int^, F4/80^+^) and engraftment were analyzed via flow cytometry.

### Flow cytometry and cell sorting.

The following antibodies were used: anti–CD45-BV510 (BioLegend), clone 30-F11; anti-CD45–Alexa Fluor 700 (BioLegend), clone I3/2.3; anti–Ly6G-PerCP (BioLegend), clone 1A8; anti–CD11b-BV510 (BioLegend), clone M1/70; anti–GR1-FITC (BioLegend), clone RB6-8C5; anti–F4/80–PE-Cy7 (BioLegend), clone BM8; anti–F4/80-APC (BioLegend), clone BM8; anti–CD115–APC/Fire 750 (BioLegend), clone AFS98; anti–CD86-BV421 (BioLegend), clone GL-1; anti–CD206-PE (BioLegend), clone C068C2; anti–NK1.1–APC-Cy7 (BioLegend), clone PK136; anti–B220-BV421 (BioLegend), clone RA3-6B2; anti–CD4–PE-Cy7 (BioLegend), clone GK1.5; anti–CD8-PerCP (BioLegend), clone 53-6.7; anti–IFN-γ–APC (BioLegend), clone XMG1.2; anti–annexin V–PE-Cy7 (Thermo Fisher Scientific); anti–annexin V–APC (BioLegend); anti–CD31–Pe-Cy5 (BioLegend), clone WM59; anti–CD45-APC (BioLegend), clone 30-F11; anti–CD34-BV421 (BioLegend), clone 561; anti–Lineage Cocktail–Pacific Blue (BioLegend), clones 17A2, RB6-8C5, RA3-6B2, Ter-119, and M1/70; and anti–CD33-PE (BioLegend), clone P67.6. Antibody cocktails were prepared in MACS buffer as described above and incubated with BM cells for at least 30 minutes at 4°C. In experiments using MitoTracker Green or MitoTracker Red (Invitrogen, Thermo Fisher Scientific), the cells were incubated at room temperature for 30 minutes, washed twice in PBS, and centrifuged at 400*g* for 5 minutes before any further addition of antibodies. In experiments using Vybrant DiI Cell-Labeling (Invitrogen, Thermo Fisher Scientific), the cells were incubated at room temperature for 20 minutes, washed twice in PBS, and centrifuged at 1200 rpm for 5 minutes. For sorting of AML-associated BMMs, BM cells were resuspended in antibody mix, and cells were sorted directly into lysis buffer. Sorted BMMs were also sorted into RPMI-1640 containing 20% FBS plus 1% penicillin-streptomycin supplemented with macrophage CSF (10 ng/mL). Sorted mitochondria-containing ABs were sorted directly into PBS. Compensations and fluorescence minus one (FMO) controls were run for each panel.

Flow cytometry was performed using a FACSCanto II flow cytometer (BD Bioscience), and cell sorting was performed on a BD FACSMelody (BD Bioscience) or an SH800S Cell Sorter (Sony). Image flow cytometry was carried out using the Amnis ImageStream^x^ Mk II (Luminex). Data were analyzed using FlowJo (TreeStar).

### qPCR.

RNA from cells was isolated using the ReliaPrep RNA Miniprep System (Promega). A real-time qPCR assay was performed with 1-step SYBR Green technology (PCR Biosystems) with QuantiTect Primer Assays (QIAGEN). After a reverse transcription step (45°C for 10 minutes), PCRs were then amplified for 45 cycles (polymerase activation at 95°C for 2 minutes followed by cycles of 95°C for 10 seconds, 60°C for 10 seconds, and 72°C for 30 seconds) on a Roche 384-well LightCycler480. mRNA expression was normalized to GAPDH using the comparative cycle threshold method, and calculations were done using the ΔΔCt method.

### ρ^0^ MN1 generation.

MN1 cells (5 × 10^6^) were cultured in DMEM containing 10% FBS plus 1% penicillin-streptomycin supplemented with mSCF (100 ng/mL), mIL-3 (10 ng/mL), hIL-6 (10 ng/mL), ethidium bromide (1 μg/mL), 2′,3′-dideoxycytidine (ddC) (200 μM), sodium pyruvate (100 μg/mL), and uridine (50 μg/mL). Every 7 days, cells were centrifuged at 1200 rpm for 5 minutes and resuspended in fresh media. On day 20, mtDNA detection was performed using qPCR as previously described (11). Briefly, DNA was extracted from MN1 and ρ^0^ MN1 cells using the GenElute Mammalian Genomic DNA Miniprep Kit (MilliporeSigma) according to the manufacturer’s protocols. Purified DNA was then analyzed via TaqMan probe ND3 for mtDNA and normalized to Tert for genomic DNA (Thermo Fisher scientific). The relative ratio of mtDNA to genomic DNA (gDNA) was calculated using the ΔΔCt method.

### Apoptosis induction and AB isolation.

To induce apoptosis, cells were treated with 5 μM cytosine arabinoside (ara-C) for 24 hours. Apoptosis was determined by annexin V–PE-Cy7 labeling (Invitrogen, Thermo Fisher Scientific) using flow cytometry. ABs were isolated via centrifugation as previously described (88). Briefly, cells were spun at 500*g* for 10 minutes. The supernatant was spun at 12,000*g* for 10 minutes, and the pellet was resuspended in either PBS or the appropriate media.

### Microscopy.

Isolated F4/80^+^ BMMs were plated at a density of 2 × 10^5^ on a μ-Plate 24-well black plate (ibidi) in 500 μL RPMI-1640 containing 20% FBS plus 1% penicillin-streptomycin supplemented with macrophage CSF (10 ng/mL) until required or in FluoroBrite DMEM containing 10% FBS plus 1% penicillin-streptomycin for immediate use. Zymosan A (*Saccharomyces cerevisiae*) BioParticles, Texas Red conjugate (Invitrogen, Thermo Fisher Scientific) were incubated for 2 hours with the BMMs according to the manufacturer’s instructions. Cells were washed twice with PBS before fixation and permeabilization (Thermo Fisher Scientific), during which LC3-FITC and DAPI were incubated with the BMMs for 20 minutes. Cells were washed twice with PBS, and 500 μL FluoroBrite DMEM was added. Cells were observed under an EVOS M5000 Imaging System (Thermo Fisher Scientific) at ×40 magnification. BMMs were plated at a density of 2 × 10^5^ cells on a μ-Plate 24-well black plate in 500 μL RPMI-1640 containing 20% FBS plus 1% penicillin-streptomycin for 24 hours. MN1 ABs were generated and isolated as described above, with the exception that ACs were incubated for 30 minutes with pHrodo Red, SE (Invitrogen, Thermo Fisher Scientific), according to the manufacturer’s instructions, before AB isolation. The media were removed from the BMMs, 500 μL FluoroBrite DMEM was added, and ABs were cultured with the BMMs for 3 hours. Cells were fixed and permeabilized (Thermo Fisher Scientific), during which LC3-FITC and DAPI were incubated with the BMMs for 20 minutes. Cells were washed twice with PBS, and 500 μL FluoroBrite DMEM was added. Cells were observed under an EVOS M5000 Imaging System at ×40 magnification (Invitrogen, Thermo Fisher Scientific). Sorted BMMs were plated at a density of 1.5 × 10^5^ cells on a μ-Plate 24-well black plate in 500 μL RPMI-1640 containing 20% FBS plus 1% penicillin-streptomycin for 24 hours. The media were changed to 500 μL FluoroBrite DMEM containing 10% FBS plus 1% penicillin-streptomycin, and BMMs were cultured with the STING inhibitor H-151 (10 μM, InvivoGen) or vehicle (PBS with 0.1% Tween-80) for 2 hours. BMMs were then incubated with pHrodo Red *E*. *coli* BioParticles (Invitrogen, Thermo Fisher Scientific) for 2 hours before imaging on a Zeiss LSM 800 Axio Observer.Z1 confocal microscope with a ×63 water objective (Carl Zeiss).

Human AML cells were transduced with a rLV.EF1.AcGFP-Mem9 lentivirus (Clontech Takara Bio Europe) for plasma membrane expression of GFP (AML-GFP). AML-GFP cells were stained with MitoTracker Red (Invitrogen, Thermo Fisher Scientific) according to the manufacturer’s protocol and with Hoechst 33342 (Thermo Fisher Scientific). Images were captured on a Zeiss LSM 800 Axio Observer.Z1 confocal microscope with a ×63 water objective (Carl Zeiss). For May-Grunwald Giemsa–stained BM smear slides, an Olympus BX51 light microscope was used.

### Cytokine array.

BMMs were isolated from Atg16l^E230+^ and Atg16l^E230–^ mice as described earlier in Methods and plated at a density of 2.5 × 10^5^ cells in 24-well plates in 200 μL RPMI-1640 containing 10% FBS plus 1% penicillin-streptomycin. Vehicle (PBS) or ABs from MN1 cells were generated as described above and added to the BMMs for 24 hours. The supernatant was removed, pooled, and centrifuged at 10,000 rpm for 10 minutes to remove any debris. Cell supernatant was used for the Proteome Profiler Mouse XL Cytokine Arrays (R&D Systems) following the manufacturer’s protocol. Cytokine membranes were analyzed using the G:BOX Chemi XRQ system (Syngene) and quantified using ImageJ (Fiji, NIH).

### In vivo STING inhibition.

Mice were treated with 25 mg/kg busulfan on day –2 and day –1 prior to tail vein injections of 1 × 10^6^ MN1-GFP cells on day 0. On days 7, 9, 11, and 13 after MN1-GFP injection, mice were i.p. injected with 200 μL H-151 (750 nmol, InvivoGen) or vehicle (PBS with 0.1% Tween-80). The animals were sacrificed on day 14 after injection, and BM was isolated as described earlier in Methods. The BM was analyzed using flow cytometry for MN1 engraftment and T cell activity. BMMs were sorted, and the RNA was extracted for qPCR analysis.

### Engraftment of AML cells into NSG mice.

Primary AML blasts (1 × 10^6^), with and without rLV.EF1.mCherry-Mito9 lentivirus transduction, were injected i.v. into nonirradiated 6- to 8-week-old NSG mice. At predefined humane endpoints, the animals were sacrificed, BM was isolated, and engraftment was determined according to expression of human CD45. Mouse BM cells were examined via flow cytometry for BMSCs (CD45^–^CD105^+^), BMMs (CD45^+^GR1^–^CD115^lo/int^F4/80^+^), and mature lymphoid cells (CD45^+^CD3^+^). The MFI mCherry intensity of each population was analyzed using FlowJo software.

### Statistics.

For statistical analysis of 2 groups, an unpaired Mann-Whitney *U* test was used. When more than 2 groups were compared, a Kruskal-Wallis test (1-way ANOVA) was performed followed by a Dunn’s post hoc test for significance using GraphPad Prism, version 7.0a for Mac OSX (GraphPad Software). Differences among group means were considered significant when the *P* value was less than 0.05.

### Study approval.

All animal work in this study was carried out in accordance with regulations established by the UK Home Office (London, United Kingdom) and the Animal Scientific Procedures Act of 1986. Nonmalignant and malignant hematopoietic cells were collected at the Norfolk and Norwich University Hospital. Studies were performed following approval from the United Kingdom Health Research Authority research ethics committee (ref. 07/H0310/146). Primary AML blasts were obtained from the BM of patients with AML after informed consent and under the approval of the UK National Research Ethics Service (LRCE, ref. 07/H0310/146).

## Author contributions

JAM, JJM, KMB, NB, and SAR designed the research. JAM, JJM, CH, RHH, EEW, and AJ performed the research. JAM, JJM, NB, SAR, and CH and carried out the in vivo work. TW and MJ provided essential reagents and knowledge and proofread the manuscript. JAM, NB, KMB, and SAR wrote the manuscript.

## Supplementary Material

Supplemental data

## Figures and Tables

**Table 1:**
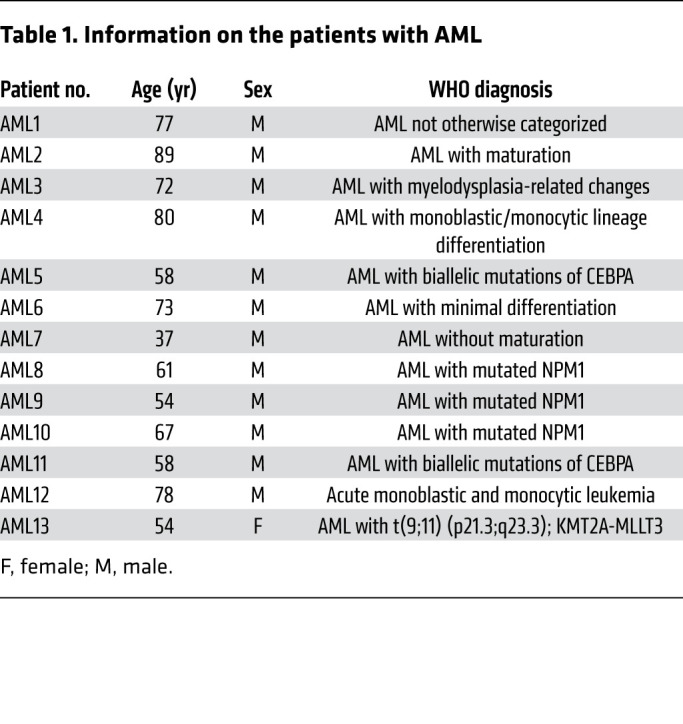
Information on the patients with AML

## Version 1 01/06/2022

In-Press Preview

## Version 2 03/01/2022

Electronic publication
