# Supplementary material for: LC3-associated phagocytosis in bone marrow macrophages suppresses acute myeloid leukemia progression through STING activation
Source: J Clin Invest. 2022 Mar 1;132(5):e153157. doi: 10.1172/JCI153157 (PMC8884913; doi:10.1172/JCI153157)
Supplement: Supplemental data [file jci-132-153157-s141.pdf]

Supplementary Figure 1.

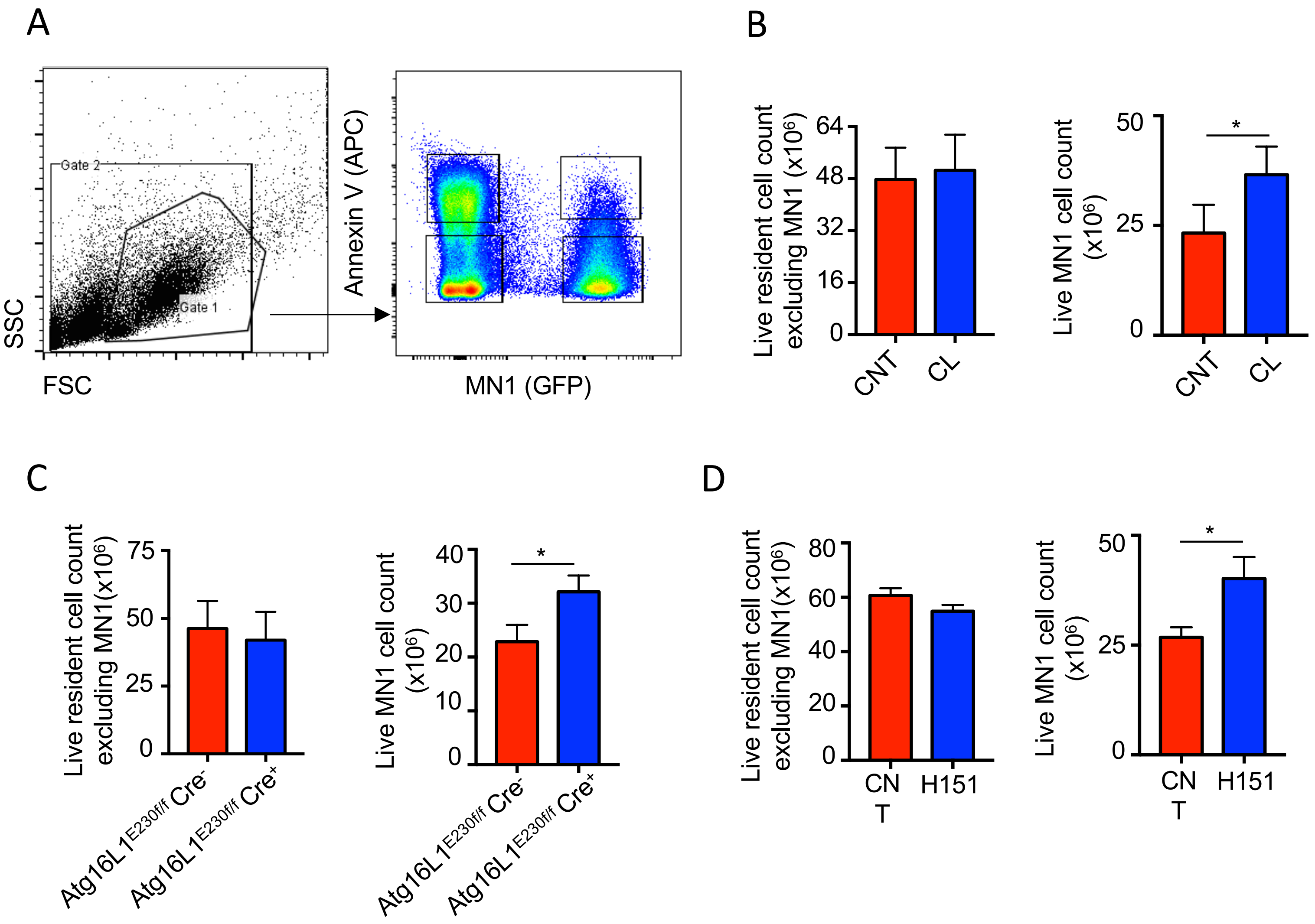

Supplementary Figure 1. Live absolute resident cell and MN1 cell counts in clodronate treated and E230 mice engrafted with AML. (A) Representative flow cytometry plots to identify live and apoptotic resident cell and MN1 cell populations by Annexin V staining from Gate 1 of the BM. Gate 2 is used to measure data presented in figure 3A. (B) Live absolute cell counts of resident cells excluding MN1-GFP and live MN1-GFP following treatment with control (CNT) or clodronate (CL) liposomes. (C) Live absolute cell counts of resident cells excluding MN1-GFP and live MN1-GFP from Atg16L1$^{E230f/f}$ Cre^{+} and Atg16L1$^{E230f/f}$ Cre^{-} mouse bone marrow after MN1-GFP injection. (D) Live absolute cell counts of resident cells excluding MN1-GFP and live MN1-GFP from CNT and H-151 treated mouse bone marrow after MN1-GFP injection.

Supplementary Figure 2.

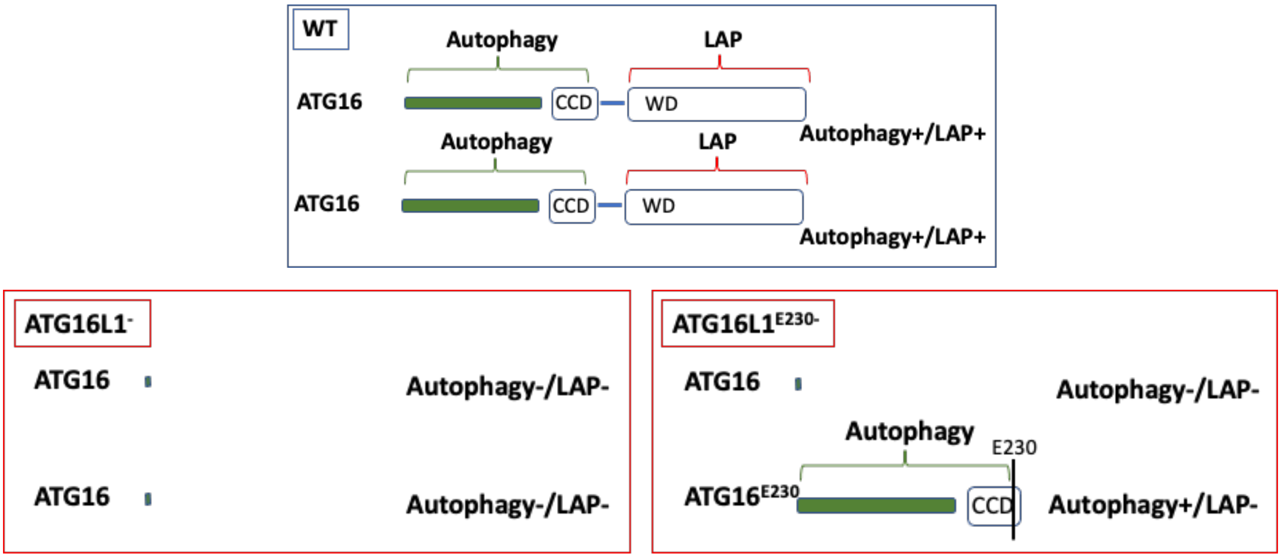

Supplementary Figure 2. Schematic of Atg16l1^{E230} mice. 2 stop codons were inserted into exon 6 immediately after glutamate E230 to preserve binding sites for WIPI2 but prevent translation of the linker and WD domain.

Supplementary Figure 3.

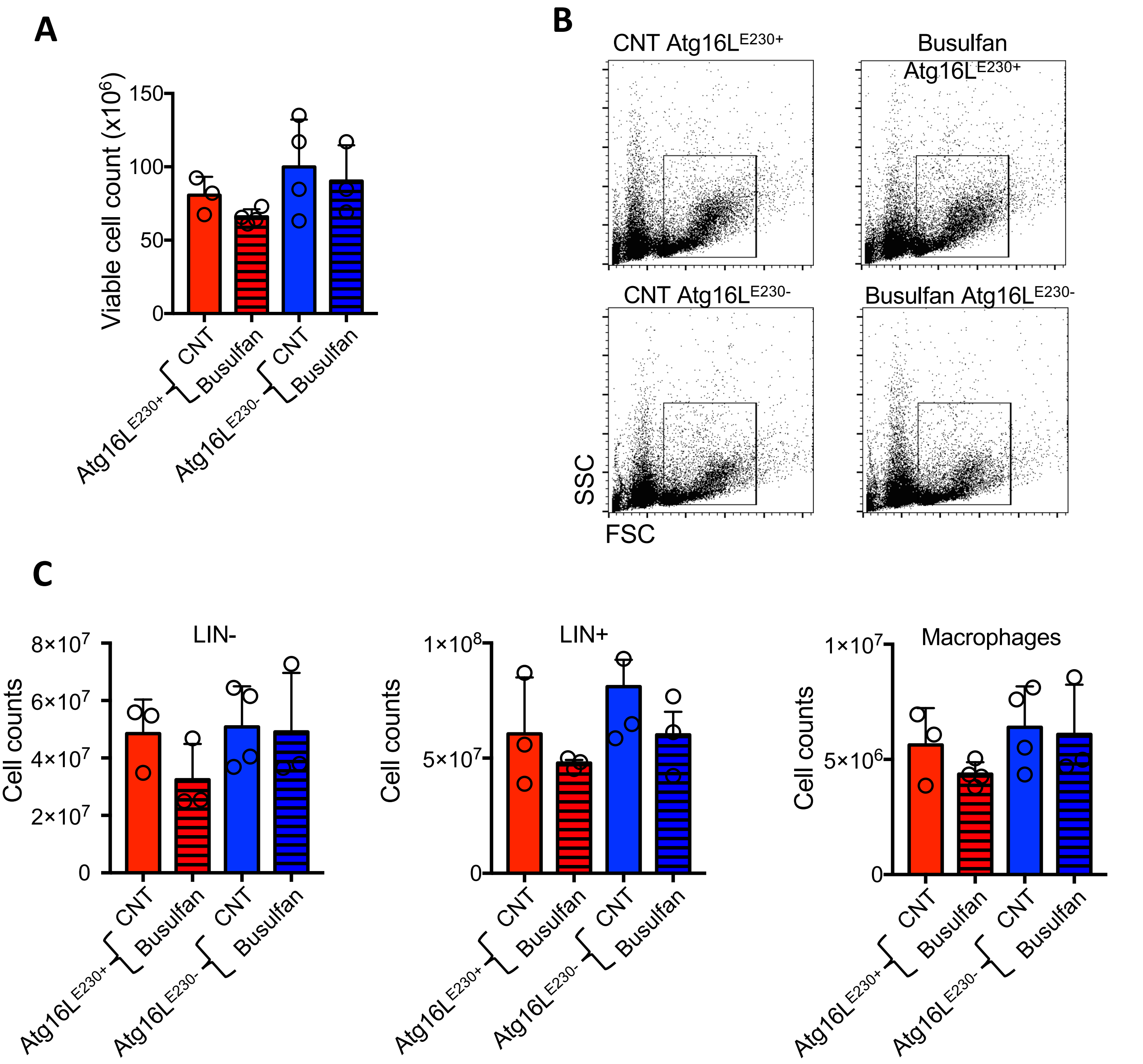

Supplementary Figure 3. Bone marrow cellularity in Atg16L^{E230-} and Atg16L^{E230+} animals with and without busulfan treatment (2-day treatment; 25mg/kg, bone marrow analyzed 14 days). (A) Total cell count of viable bone marrow after extraction and trypan blue exclusion. (B) Flow plots of bone marrow cellularity between groups (C) Cell counts of lineage negative cells, lineage positive cells and macrophages analyzed via flow cytometry.

Supplementary Figure 4.

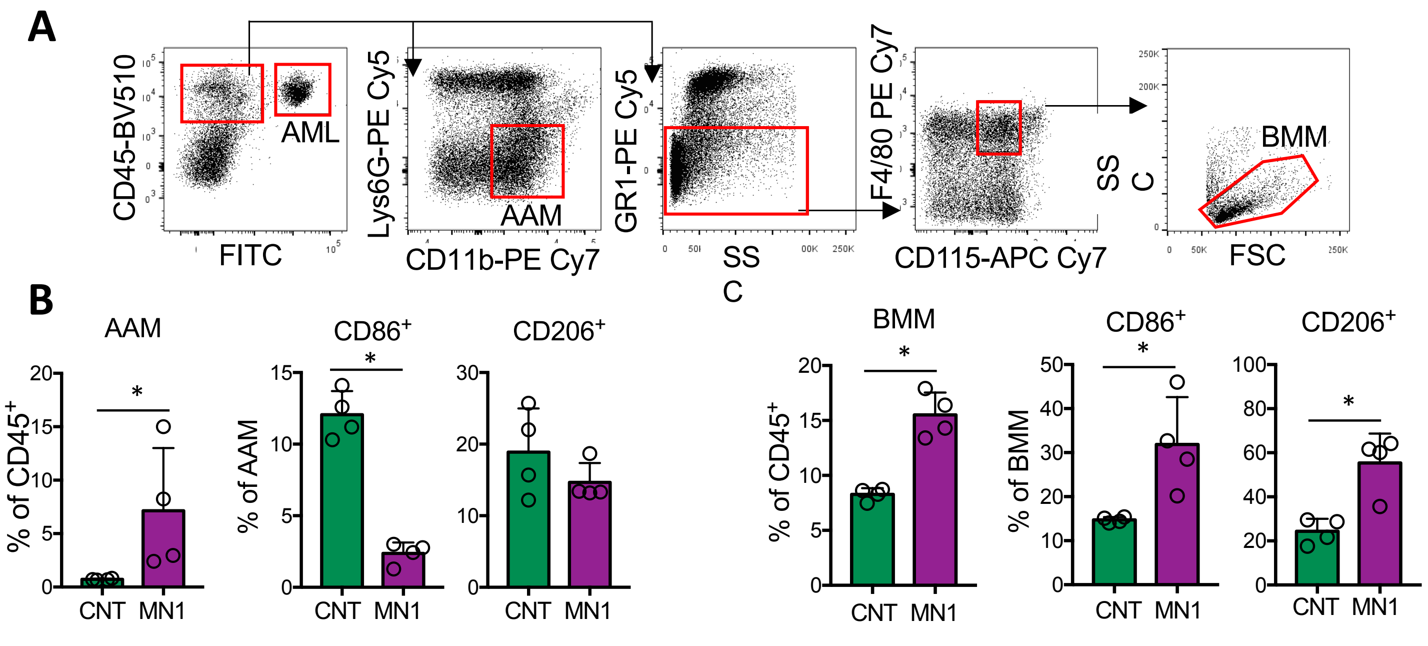

Supplementary Figure 4. (A) Representative flow plot and gating strategy for AAM (CD45^{+}, Lys6G^{-} and CD11b^{+}) and BMM (CD45^{+}, GR1^{-}, CD115$^{LOW/INT}$ and F4/80^{+}). (B) MN1 cells (1x10^{6}) or vehicle (PBS) were injected into busulfan treated C57/BL6 mice, and the BM harvested 14 days post injection. The percentage of AAM cells in the BM as well as the percentage of CD86 and CD206 expressing AAM cells was analysed via flow cytometry. The percentage of BMM cells in the BM as well as the percentage of CD86 and CD206 expressing BMM cells was analysed via flow cytometry.

Supplementary Figure 5.

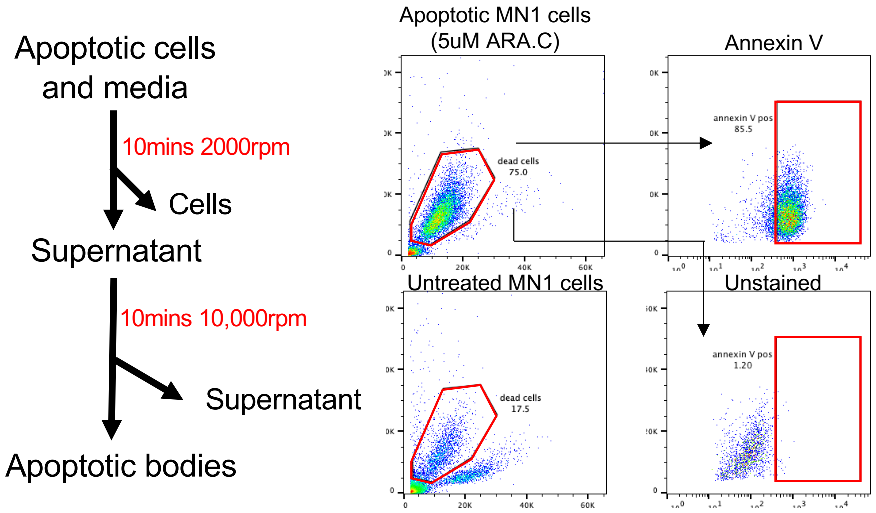

Supplementary Figure 5. Isolation of MN1 derived apoptotic bodies and identification of annexin V positive staining.

Supplementary Figure 6.

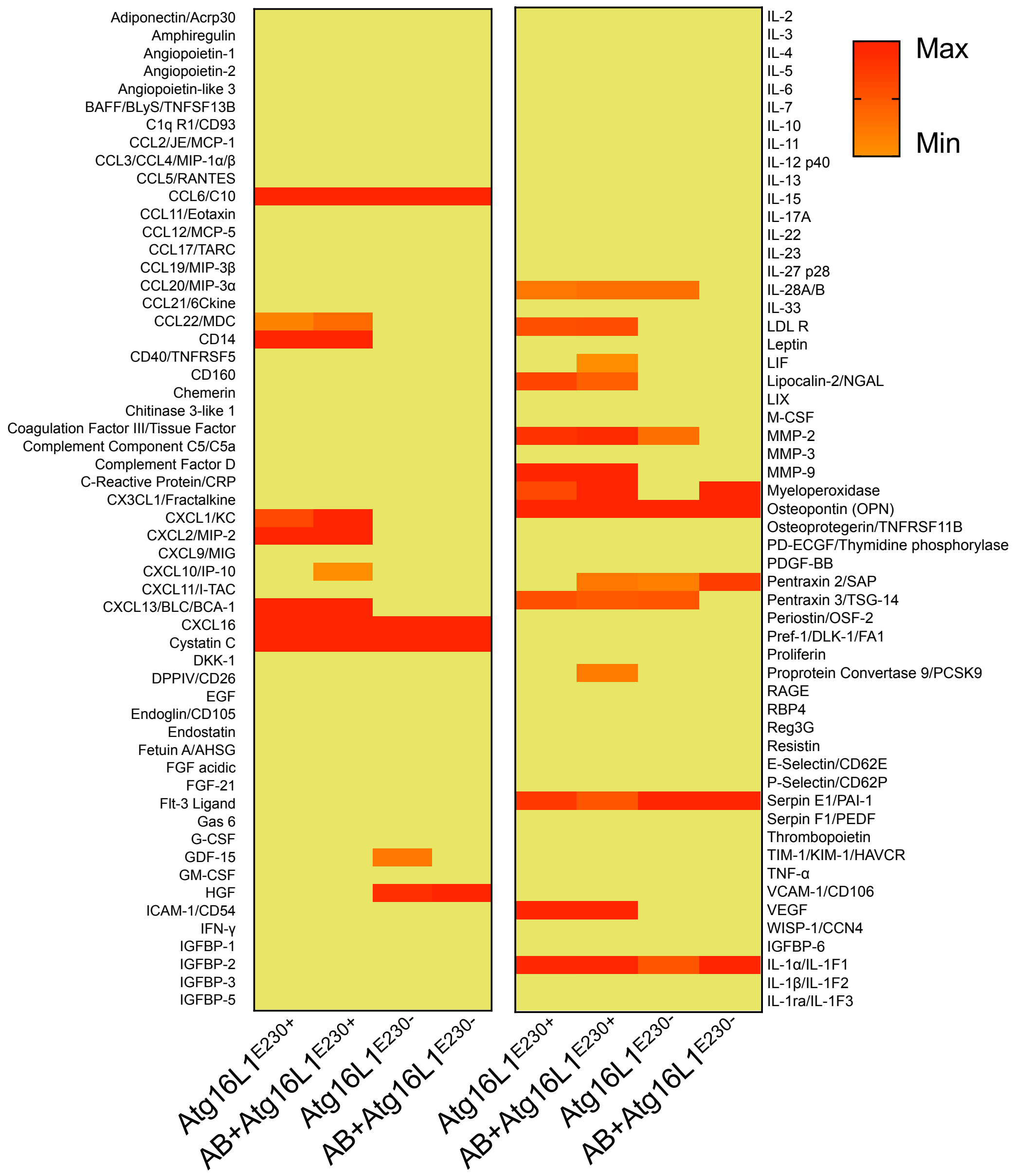

Supplementary Figure 6. Heatmap of all cytokine analytes from BMDM isolated from Atg16L^{E230+} and Atg16L^{E230-} mice, with and without treatment of apoptotic bodies isolated from MN1 cells.

Supplementary Figure 7.

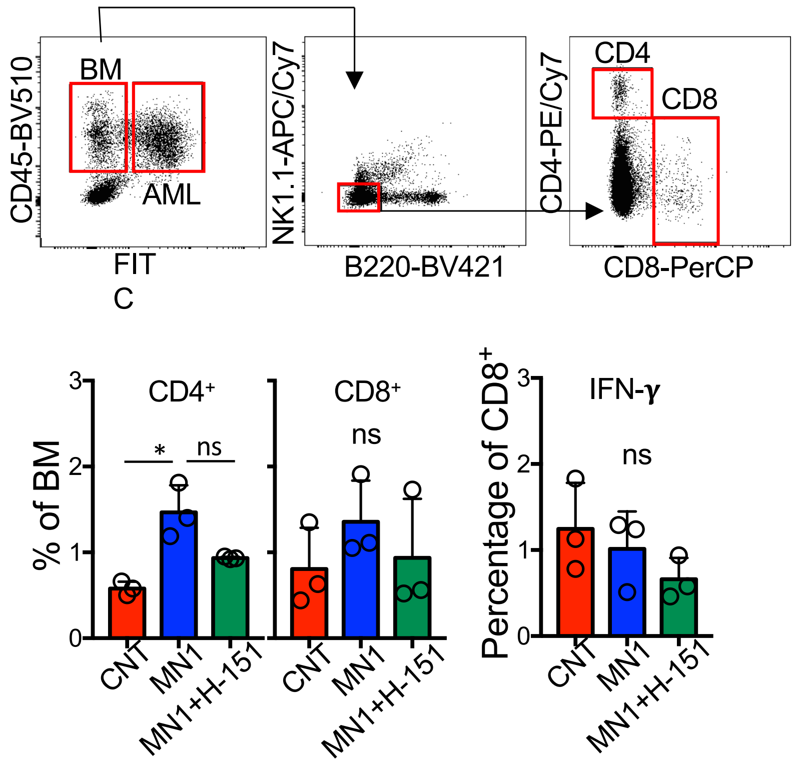

Supplementary Figure 7. Representative flow plot and gating strategy for CD4 and CD8 T-cell populations. The percentage of $CD4^+$ and $CD8^+$ cells in the BM as well as the percentage of IFN-**γ** expressing $CD8^+$ cells for C57/BL6 mice engrafted with either MN1 cells treated with H-151 or vehicle and control mice.

Supplementary Figure 8.

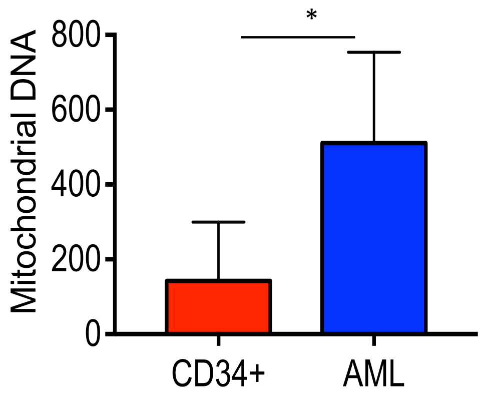

Supplementary Figure 8. Mitochondrial DNA quantification in AML and $CD34^{+}$ cells.

Supplementary Figure 9.

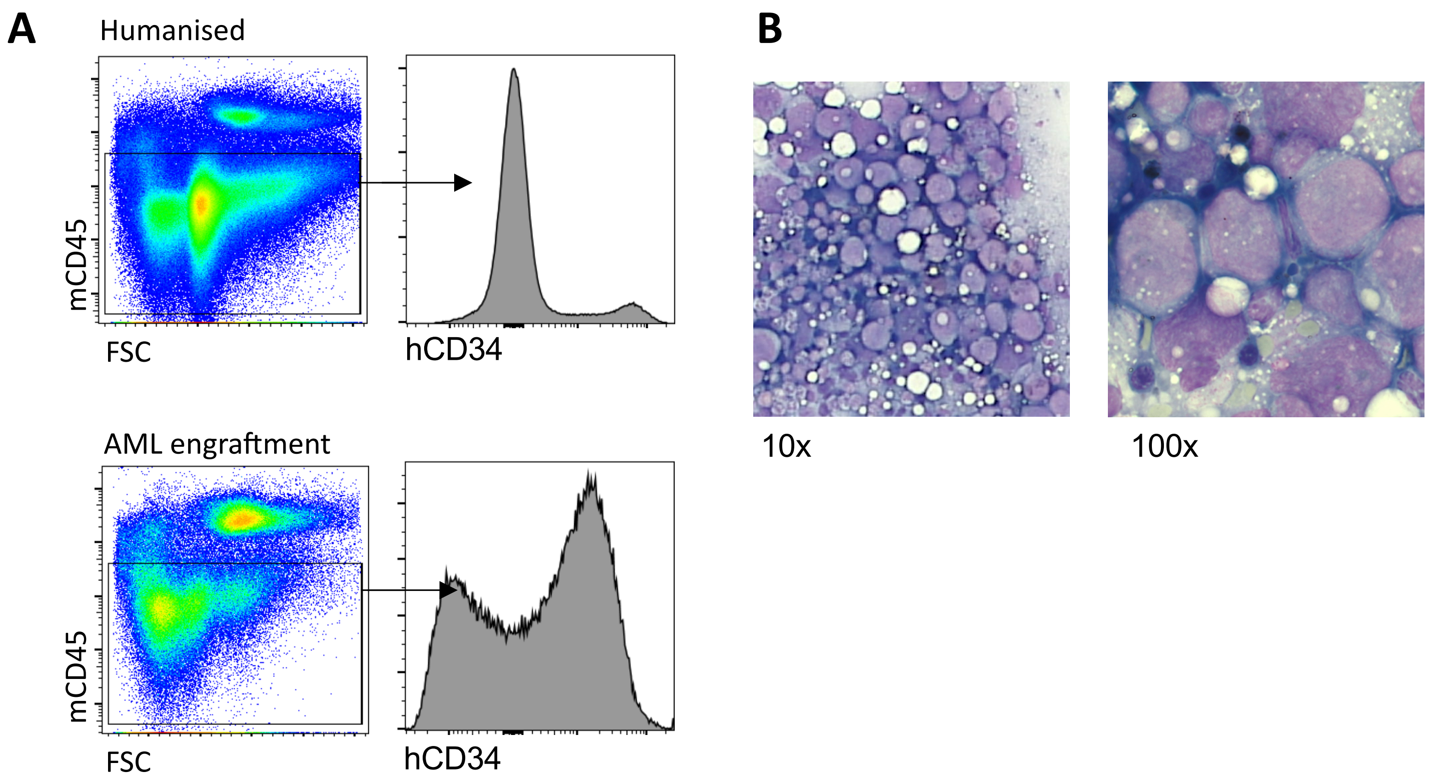

Supplementary Figure 9. (A) Identification of differences in humanized NSG mice models compared to human AML engraftment in NSG mice via flow cytometry for $CD34^+$ cells. (B) Bone marrow of human AML engrafted NSG mice was smeared onto slides and following H&E staining light microscopy was perfomed to identify AML blasts.

Supplementary Figure 10.

A

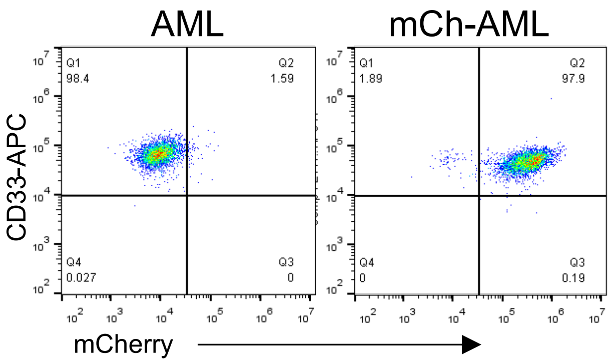

B

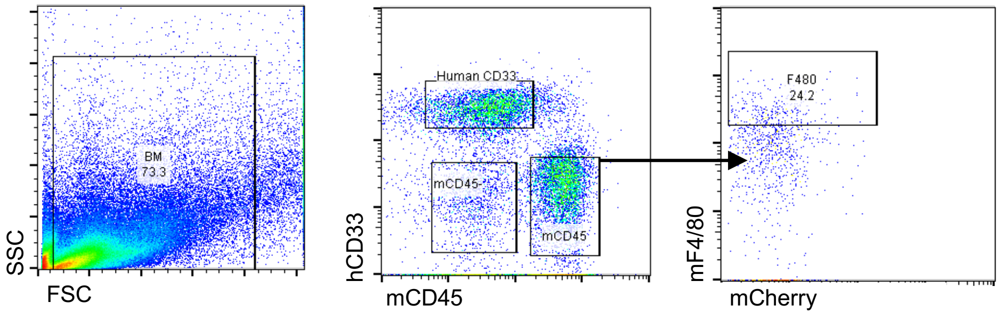

Supplementary Figure 10. (A) mCherry fluorescence in AML and mCh-AML cells identified via flow cytometry. (B) Representative flow plot to identify mCherry positive macrophages (mCD45+, F4/80+, mCherry+) in NSG animal model AML engraftment experiment.

Supplementary Table 1

Number	Age	Sex	WHO diagnosis
AML1	77	M	AML not otherwise categorised
AML2	89	M	AML with maturation
AML3	72	M	AML with myelodysplasia-related changes
AML4	80	M	AML with monoblastic/monocytic lineage differentiation
AML5	58	M	AML with biallelic mutations of CEBPA
AML6	73	M	AML with minimal differentiation
AML7	37	M	AML without maturation
AML8	61	M	AML with mutated NPM1
AML9	54	M	AML with mutated NPM1
AML10	67	M	AML with mutated NPM1
AML11	58	M	AML with biallelic mutations of CEBPA
AML12	78	M	Acute monoblastic and monocytic leukaemia
AML13	54	F	Acute myeloid leukaemia with t(9;11)(p21.3;q23.3); KMT2A-MLLT3

Supplementary Table 1. AML patient information.
